# Deficiency of metabolic regulator FGFR4 delays breast cancer progression through systemic and microenvironmental metabolic alterations

**DOI:** 10.1186/2049-3002-1-21

**Published:** 2013-11-25

**Authors:** Yongde Luo, Chaofeng Yang, Min Ye, Chengliu Jin, James L Abbruzzese, Mong-Hong Lee, Sai-Ching J Yeung, Wallace L McKeehan

**Affiliations:** 1 Center for Cancer and Stem Cell Biology, Institute of Biosciences and Technology, Texas A&M Health Science Center, 2121 W. Holcombe Blvd., Houston, TX 77030-3303, USA; 2 IBT Proteomics and Nanotechnology Laboratory, Institute of Biosciences and Technology, Texas A&M Health Science Center, 2121 W. Holcombe Blvd, Houston, TX 77030-3303, USA; 3 Department of Animal Resources, Georgia State University, Atlanta, GA 30303, USA; 4 Department of Gastrointestinal Medical Oncology, The University of Texas MD Anderson Cancer Center, Houston, TX 77030, USA; 5 Department of Molecular and Cellular Oncology, The University of Texas MD Anderson Cancer Center, Houston, TX 77030, USA; 6 Department of Emergency Medicine & Department of Endocrine Neoplasia and Hormonal Disorders, The University of Texas MD Anderson Cancer Center, Houston, TX 77030, USA

**Keywords:** Adipokines, Breast cancer, endocrine fibroblast growth factor (eFGF), Fibroblast growth factor receptor (FGFR), Inflammatory response, Metabolism, Microenvironmental and systemic effects, Transforming growth factor alpha (TGFα)

## Abstract

**Background:**

Endocrine FGF21 and FGF19 target adipocytes and hepatocytes through betaKlotho (KLB) and FGFR tyrosine kinases effecting glucose, lipid and energy metabolism. Both factors alleviate obesity and metabolic abnormalities which are contributing factors to breast tumor progression. Genomic manipulation of hepatic FGFR4 has uncovered roles of endocrine FGF signaling in both metabolic and cellular homeostasis. Here we determined whether systemic and microenvironmental metabolic alterations caused by the FGFR4 deficiency affect tumorigenesis in breast where FGFR4 is negligible. Breast tumors were induced in the bigenic mice with ablation of FGFR4 and overexpression of TGFα that activates Her2 in the ductal and lobular epithelium surrounded by adipocytes. Mammary tumorigenesis and alterations in systemic and breast microenvironmental metabolic parameters and regulatory pathways were analyzed.

**Results:**

Ablation of FGFR4 had no effect on cellular homeostasis and Her2 activity of normal breast tissue. However, the absence of FGFR4 reduced TGFα–driven breast tumor incidence and progression and improved host survival. Notable increases in hepatic and serum FGF21, ileal FGF15/19, adiponectin and adipsin, and decreases in systemic Fetuin A, IGF-1, IGFBP-1, RBP4 and TIMP1 were observed. The ablation affected adipogenesis and secretory function of adipocytes as well as lipogenesis, glycolysis and energy homeostasis associated with the functions of mitochondria, ER and peroxisomes in the breast and tumor foci. Treatment with a chemical inhibitor of NAMPT involved in the pathways inhibited the growth and survival of breast tumor cells and tumor-initiating cell-containing spheres. The FGFR4 ablation also caused elevation of inflammatory factors in the breast.

**Conclusions:**

Although the primary role of FGFR4 in metabolism occurs in hepatocytes, its ablation results in a net inhibitory effect on mammary tumor progression. We suggest that the tumor-delaying effect of FGFR4 deficiency may be in large part due to elevated anti-obesogenic FGF21 that triggers tumor-suppressing signals from both peripheral and breast adipocytes. The predominant changes in metabolic pathways suggested roles of metabolic effects from both peripheral and breast adipocytes on metabolic reprogramming in breast epithelial cells that contribute to the suppression of tumor progression. These results provide new insights into the contribution of systemic and microenvironmental metabolic effects controlled by endocrine FGF signaling to breast carcinogenesis.

## Background

Fibroblast growth factor receptors (FGFRs) are transmembrane receptor tyrosine kinases that sense extracellular environmental cues and trigger adaptive cellular responses that include growth, survival and metabolic programming [1,2]. They are encoded by four genes, namely *FGFR1*, *2*, *3 and 4*, with a number of splice isoforms. In association with co-factor heparan sulfate (HS) proteoglycan motifs and tissue-specific HS-binding and matrix-controlled FGF ligands, these tyrosine kinases play paracrine and autocrine roles in embryonic development and adult tissue homeostasis through control of cell proliferation, survival, migration and differentiation [3-5]. Like epidermal growth factor receptor (EGFR) and human epidermal growth factor receptor-2 (Her2), aberrations in the components of the canonical FGF signaling pathway contribute to hyperplastic growth and tumorigenesis [6-9].

However, in the presence of tissue-specific transmembrane co-receptor Klotho (KL) and betaKlotho (KLB) whose significant expression is limited to tissues involved in systemic metabolism, and the KL/KLB-binding endocrine FGFs (eFGFs) that include FGF19 (mouse FGF15), 21 and 23, the same FGFR tyrosine kinases play distinct roles in maintaining local and systemic homeostasis of lipid, glucose, energy and mineral metabolism. This shift occurs without an equal effect on mitogenic and growth-promoting activities observed with canonical FGFs [10-14]. The integration of tissue-specific KL/KLB with the FGFR signaling complex appears to be the key to this distinction [11,15,16]. KLB forms a binary complex with FGFRs in metabolic tissues that confers high affinity for the eFGFs [10,17], and in some cases, prevents high affinity binding of canonical FGFs [15]. The inter-organ cross-talking endocrine axis from hepatic FGF21 to adipocyte FGFR1-KLB is a stress-responsive pathway leading to the correction of deranged glucose, lipid and energy metabolism that benefit the organism during conditions of stress-induced pathologies such as obesity, diabetes, fatty liver diseases, malignant transformation and insults from toxins and infection [13,17-20]. Some of these pathologies are also risk factors for tumorigenesis [21-24]. Ablation or overexpression of FGF21 appears to have no significant and direct effect on cell growth and cellular structures in cells and tissues [14,25-27], and its effects on metabolism are largely mediated directly by FGFR1-KLB in adipocytes. The biological significance of the distinction as well as the association of the canonical cellular and non-canonical metabolic signal pathways mediated by a same FGFR tyrosine kinase in respect to growth and metabolism is presently unclear. They provide novel opportunities for the treatment of obesity, type 2 diabetes, hypophosphatemia and diverse types of cancer where aberrant metabolism is a complicating contributor.

Compared to other FGFRs having a wider tissue-expression pattern and high mitogenic potential, FGFR4 is highly expressed in the hepatocytes, and to a lesser extent or in a short time window in the muscle or during muscle development [28-32]. When compared side by side with other FGFRs under the same cellular condition, the specific kinase activity of wild-type (WT) FGFR4 and its activation of downstream signaling relays in the presence of canonical FGFs is quantitatively less, although its intracellular kinase domain through truncation or mutation appears to be equally active [33-35]. However, in the presence of resident KLB in hepatocytes, FGFR4 is highly and specifically activated by ileum-derived postprandial FGF19/15 and negatively regulates bile acids synthesis and lipid metabolism with far-reaching indirect effects on energy metabolism [30,36,37]. The presence of KLB appears to limit the functions of WT FGFR4 to metabolic regulation rather than canonical cellular effects. Consistently, ablation of KLB, FGF15 or FGFR4 causes hepatic deregulation of bile acid synthesis without any apparent disruption of tissue architecture and cellular homeostasis [30,37-39]. In addition, mice deficient in FGFR4 exhibit mild systemic alterations in metabolism that are related to obesity and diabetes [36].

The significant expression of KLB is largely limited to tissues involved in endocrine-controlled metabolic functions that include mainly the adipose tissue and liver, and does not overlap with the expression of FGFRs in other tissues [10,17,32,40,41]. It is lost in the progression of tumorigenesis such as hepatocarcinogenesis, and similar to its homologue KL exhibits inhibitory effects on the canonical growth-promoting functions of the FGFRs [11,14-16,42]. The epithelial compartment of tissues other than liver, adipose tissue and kidney appears to be devoid of significant levels of KL/KLB. Some studies of FGFR4 in mitogenicity and tumor development suggest that the role of FGFR4 is insignificant or even suppressive in tumorigenesis [11,43-46]. Some reports indicate variants or overexpression of FGFR4 in tumors such as the lung and pituitary where KLB is either not expressed at significant levels in the parent tissues, or downregulated or lost [47-49]. These inconsistencies may be due to variable degrees of canonical cell autonomous roles of overexpressed or mutant FGFR4 in the absence of KLB, or indirect microenvironmental effects triggered by systemic metabolic effects governed by the FGFR4-KLB partnership and eFGFs. Although aberrant canonical FGF signaling is commonly associated with the abnormal cellular activities in tumorigenesis, the KLB-mediated functional switch of FGFRs to metabolic control provides a unique model to dissect the canonical cellular and metabolic roles of FGF/FGFR signaling in tumorigenesis.

In addition to hepatocytes, the mature adipocytes are one of the major cell types that express KLB. In contrast to hepatocytes, FGFR1 rather than FGFR4 is the major FGFR isotype with KLB that mediates the metabolic response of adipose tissue to eFGFs. Although adipocytes comprise body fat depots as an independent tissue, they are dispersed throughout the body among organs to different extents and increase with obesity. The breast tissue is comprised of mostly fat interspersed with ductal lobes that successively branch deeply into numerous smaller lobular ducts until the formation of terminal units that underlies basic breast function. It is ducts, lobules and terminal units formed by the luminal epithelium of cuboidal to columnar cells where breast cancers are thought to mostly arise. Adipocytes surrounding lactiferous ducts, lobules and terminal units are thought to be of major microenvironmental impact on the function and fate of these units both in normal physiology and during tumor progression. Metabolic abnormalities, particularly those associated with obesity, have been shown as a major risk factor for breast tumorigenesis. The interplay between the adipose and ductal compartments underlies an excellent model to study the effect of both local and systemic changes in metabolism on mammary tumorigenesis.

In our previous reports, we showed that both FGF21 and FGF19 have profound beneficial effects in normalizing local and systemic metabolic parameters through targeting adipose tissues and liver, and are excellent candidates or pathway targets for treatment of obesity, diabetes and metabolic disorders [13,17,18,20]. In this report, we examined the development of spontaneous breast cancer driven by overexpression of transforming growth factor (TGF)α in the luminal epithelial cells of mouse breast [8,9] in a genetic background of FGFR4 deficiency. We found a negative impact of systemic and microenvironmental metabolic alterations resulting from this deficiency that raises systemic FGF21 on the malignant transformation of breast epithelial cells, which normally do not express FGFR4.

## Methods

### Mouse genetic manipulation and animal care

The MMTV-TGFα transgenic mouse strain (referred to as Tg thereafter), FVB.Cg-Tg(MMTVTGFΑ)254Rjc/J, was obtained from The Jackson Laboratory (Bar Harbor, ME, USA), and back-crossed to C57BL/6 J for more than five generations. The whole-body FGFR4 knockout C57BL/6 J mice (FGFR4^−/−^, referred to as KO thereafter) was established as previously described. The FGFR4^−/−^: MMTV-TGFα bigenic mice (referred to as KO-Tg thereafter) were generated by crossing C57BL/6 J Tg mice with the KO mice. Female animals with appropriate genotypes were randomized according to age and/or body weight into control and treatment/study groups.

All mice were housed in the Program of Animal Resources in the Institute of Biosciences and Technology, Texas A&M Health Science Center, and were maintained in accordance with the principles and procedure of the *Guide for the Care and Use of Laboratory Animals*. All experimental procedures were approved by the Institute of Biosciences and Technology Institutional Animal Care and Use Committee (IBT IACUC) with protocols #08058 entitled *The Impact of Obesity and Obesity Treatment in Cancer* and #10022 entitled *BetaKlotho-FGFR in the liver*.

### Genotyping

Mouse tail DNA was used for genotyping. Primer pair 5′-AGTTCTGCTTCCATGGAACC and 5′-TGATGATAAGGACAGCCAGG was used for *TGFα* transgenic mice. Primer pair 5′-ACCAACACTGGAGCCTGGT and 5′-TGGCAGACTTCTGCTCCTT was used for WT *FGFR4* mice, and the latter primer was used together with a primer 5′-ATCGCCTTCTATCGCCTTGACGA from the *Neo* gene for the FGFR4^−/−^ mice [39].

### Tumor and tissue sample harvesting

Mice were sacrificed, breast tumors and breasts were removed, and a complete autopsy for all breasts and tumors in each mouse was performed. The samples were evaluated by a pathologist in a blinded manner on the basis of H&E-stained sections, and the breast alterations were classified as hyperplasia, adenoma and duct carcinoma. The autopsy samples of lymph node, lung, liver and brain were evaluated histopathologically for the presence of metastases.

### Tissue processing and immunohistochemistry

Breasts and breast tumors were removed from mice and fixed in 4% paraformaldehyde (PFA)-PBS for 4 h. Fixed tissues were processed for ethanol dehydration and paraffin embedding as described [50]. Paraffin-embedded tissue blocks were serially sectioned and slide-mounted. The sections (5 μm) were deparaffinized and rehydrated before staining with H&E reagents, or primary and secondary antibodies for immunohistochemistry (IHC) as indicated [50]. Ki67 and 5-bromo-2′-deoxyuridine (BrdU) staining for mitotic index were done according to the manufacturer’s protocol (Sigma-Aldrich, St Louis, MO, USA). The section was then counterstained with hematoxylin and mounted with 1,1′-(1,4-phenylenebis(methylene))bis-pyridiniudibromide (DPX) media. The slides were analyzed by a pathologist and photographed digitally by light microscopy.

### Breast tumor incidence

The tumor incidence in the age-matched KO-Tg and Tg mice was defined as the percentage of mice with tumor(s). The palpable breast tumor foci found in each mouse in both groups were further confirmed by pathological examination on tissue sections under microscope, and the incidence rate of breast tumor was calculated as percentage of the whole population in each group monthly (Tg, 218 mice; KO-Tg, 206 mice). Tumor multiplicity defined as number of breasts having tumor per mouse was also recorded monthly for the duration of the experiments. Tumor size was measured with a gauge. Mice were sacrificed if the tumor load was excessive.

### Mouse survival analyses

The life span of mice was the duration between the date of birth and the date of death or mandatory sacrifice due to tumor burden or illness. The curves for the rates of overall and breast tumor-specific survival in both the FGFR4-deficient (216 mice) and WT mice (245 mice) with TGFα overexpression were constructed using the Kaplan-Meier method with the log-rank test. Overall survival was expressed as the number of months to the date of death or sacrifice in both groups suffering from illnesses as a result of TGFα overexpression and/or FGFR4 deficit. The rate of breast tumor-specific survival was defined as the number of months from diagnosis of specifically the breast cancer to the date of death or sacrifice. Differences in the frequencies of the basic characteristics, clinical parameters, and subtypes were statistically analyzed using either the chi-square test or Fisher’s exact test in cases when the expected values of any of the tumors were <5.

### Quantitative gene expression analyses

Excised breasts, breast tumor tissues, ileum and liver tissues were snap-frozen in liquid nitrogen and stored at −80°C until use. Total RNA was isolated using Ultraspec RNA Isolation reagents (Biotecx Laboratories, Houston, TX, USA). Equal amounts of total RNA from five to twelve mice were either pooled or individually used, and then 1 μg was subjected to the reverse transcription with random hexamer primer. Relative gene expression was measured by real-time PCR, and data presentation and statistical analyses were as described [17]. The pathway-focused quantitative PCR array analyses of adipogenesis, fatty acid metabolism, glucose metabolism and mitochondrial energy metabolism were performed according to the manufacturer’ s protocols (SABiosciences-Qiagen, Valencia, CA, USA), in each of which 84 predominant genes were probed. Quantitative PCR was then used to confirm the significantly altered mouse genes revealed in the array analyses. The expression levels were normalized to the β-actin in each tissue, and were then expressed as changes related to those of the WT samples, which is assigned as an arbitrary unit of 1. In addition, primer pair CGATGAGCTTGGAGAAATCA GTAGTCCATGGGCACTGTTG was used for quantitation of *Acly* expression, GAACTTGTGCAGGTTGGATG and TGCATCTCCTTTCTCTCCCT for *adiponectin*, CCACGCCAACTGTACCTATG and ATCCACTGCCATTGAACGTA for *EGFR1* (*Her1* or *ErbB1*), TCATCATTGCAACTGTGGTG and ATCTTCTGTCGCCTTCGTTT for *Her2* (*ErbB2*), GGCAAGATATACGGGCTGAT and GATGGTGCTTCATGGATCTG for *FGF15* (human *FGF19*), TTCAAATCCTGGGTGTCAAA and CAGCAGCAGTTCTCTGAAGC for *FGF21*, CTGAAGGAGGGTCATCGAAT and GTCCAGGTCTTCCACCAACT for *FGFR1*, CACCACGGACAAAGAGATTG and TGTCAACCATGCAGAGTGAA for *FGFR2*, AGATGCTGAAAGATGATGCG and ATGATGTTCTTGTGCTTGCC for *FGFR3*, GCTCGGAGGTAGAGGTCTTG and TGTTGTCCACGTGAGGTCTT for *FGFR4*, 5′-GGCGAATTATAGTGCTGCAA and 5′-TGGTGTTGCAATGAATGTTG for *HSD17B4*, 5′-CAGAGAAGGAGGAGGTGAGG and 5′-CAGCACCTGCCTTAAGTTGA for *KLB*, AGCTCTTCCTCATGGCTGTT and TTTGCCAGTTCCTCCAGATA for *IFNγ*, TGTATCTCTCTGAAGGACTCT and TGTGCAATGGCAATTCTGATT for *IL6*, AATGTCTCCTTCGGTTCTGG and CAAGGCCATTGGTTACAACA for nicotinamide phosphoribosyltransferase (*NAMPT)*, and CCTCCCTCTCATCAGTTCTAT and CAGAGAGGAGGTTGACTTTCT for *TNFα*.

### Immunoblotting and antibody array analyses

Frozen tissues were ground with mortar and pestle in liquid nitrogen, and the powdered materials were further lysed either in 1 × SDS sample buffer for direct SDS-PAGE separation and western blotting analyses, or in modified cold radioimmunoprecipitation assay (RIPA) buffer for antibody array analyses for mouse phospho-RTK according to the manufacturer’ s protocol (R&D Systems, Inc. Minneapolis, MN, USA). Protein concentration was determined by the Bradford protein assay (Bio-Rad, Hercules, CA, USA) using bovine gamma globulin as the standard.

### Serum collection and adipokine array analyses

Fresh blood was collected from bleeding through the jugular vein, and after coagulation for 1 h at room temperature serum was prepared by low-speed centrifugation for 20 minutes. Hemolysis was not observed. The sera obtained from blood samples were frozen after protein concentration was determined at −80°C until further analysis. Equal amounts of sera according to protein content were used for antibody array analyses for mouse adipokines (R&D Systems, Inc.).

### Fecal bile acids analysis

The collection of feces and extraction of bile acids from feces have been described previously [30]. Quantitative analyses of bile acids content were done by the enzyme cycling method according to the Bile Acid Assay protocol (Diazyme Laboratories, Poway, CA, USA).

### Oral glucose tolerance test (OGTT)

After an overnight fast, body weight and age-matched KO-Tg and Tg mice were randomized for glucose administration at 1 g/kg body weight by gastric gavage. Blood samples were collected, and the glucose level was determined by glucometer at 0, 15, 30, 60 and 120 minutes after delivery of the glucose load.

### Intraperitoneal insulin tolerance test (IPITT)

After 6-h fasting, mice were randomized for intraperitoneal injection of insulin at 0.75 units/kg body weight. Blood samples were collected for measurement of the glucose level at 0, 15, 30, 45, 60, 90 and 120 minutes after the insulin injection.

### Body weight monitoring

Body weights of KO-Tg and Tg groups with 216 and 245 mice, respectively, were recorded every week after birth for the duration of the experiments.

### Breast tumor cell growth and tumor-sphere formation

Solid breast tumor nodes were freshly isolated from mice, minced into pieces smaller than 1 mm in diameter in serum-free DMEM-F12 medium, and further dissociated with 0.1 mg/ml each of collagenase and hyaluronidase for 2 h at 37°C. The digested mixtures were passed through a 40-μm cell-strainer mesh tube (BD Biosciences, San Jose, CA, USA). The separated single cells in infiltrates were collected by centrifugation and cultured for one day in medium supplemented with 10 ng/ml FGF2, 10 ng/ml EGF, 1 μg/ml insulin and 0.5% BSA. Adherent cells were dissociated from the culture dish by pronase, and reseeded at 3 × 10^3^ cells/well into 6-well Ultra Low Cluster Plates (Corning Inc. Corning, NY, USA). After 3 days, a NAMPT inhibitor FK866 or cytotoxic Triptolide (Cayman Chemical, Ann Arbor, MI, USA) at 5 μM was added. Development of tumor cell spheres was counted after 2 weeks. Adherent cells at 1.5 × 10^4^ were also treated with these inhibitors for 5 days in adherent cell culture plates, and the number of cells that survived after 6 days was counted. Independent experiments were performed at least three times.

### Human breast cancer tissue microarray

Tissue microarrays of 48 cases of breast cancer, with 36 matched metastatic breast cancer and 12 normal tissue (US Biomax Inc. Rockville, MD, USA) (http://www.biomax.us/tissue-arrays/Breast/BRM961) were assessed by IHC for expression of NAMPT with an anti-NAMPT (PBEF) antibody (Santa Cruz Biotechnology, Dallas, TX, USA) as described [50]. The sections were counterstained with hematoxylin and examined by a pathologist.

### Statistical analysis

Kaplan-Meier survival curves were made using GraphPad Prism 4 (GraphPad Software, San Diego, CA, USA) and analyzed using the Mantel-Cox log-rank test. Experiments were reproduced for three times independently with triplicates within each experiment. A representative of three or more experiments may be shown in micrographs. Where indicated, the mean and SD is shown with statistic analyses by the Student unpaired *t*-test between two groups, or one-way analysis of variance (ANOVA) between multiple groups. Statistical significance is denoted as follows: ^*^P <0.05; ^**^P <0.01.

## Results

### The FGFR4 deficiency reduces breast tumor incidence

**Figure 1:**
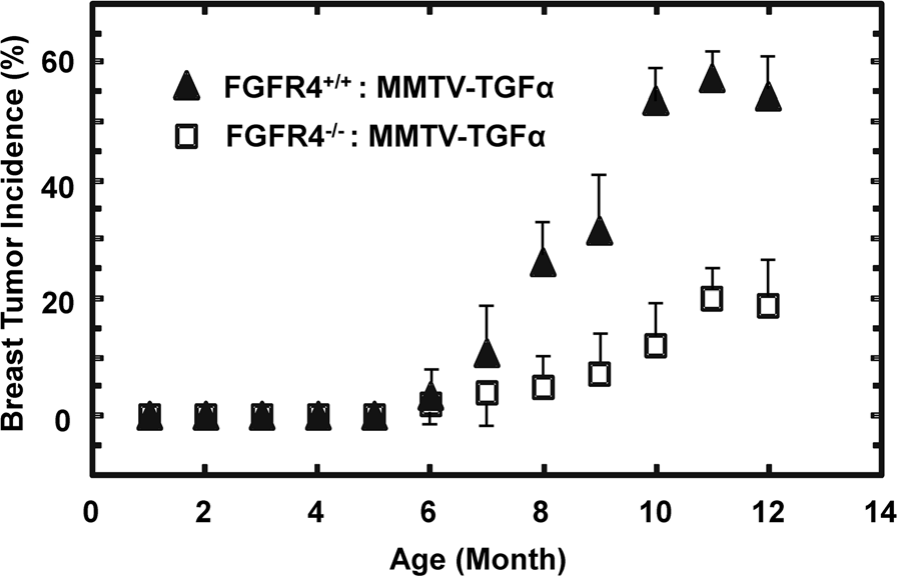
**Fibroblast growth factor receptor (FGFR)4 deficiency reduced mammary tumor incidence.** The age-matched C57BL/6 J KO-Tg and Tg mice as indicated were examined for the presence of palpable breast tumor foci that were further confirmed by pathological examination on tissue sections under microscope. The incidence rate of breast tumor was determined monthly as the percentage of the whole population in each group (Tg, 218 mice; KO-Tg, 206 mice). Experimental data from three cohorts are expressed as mean ± SD; *P* <0.05. KO, FGFR4 knockout; Tg, MMTV-TGFα transgenic mouse.

Consistent with a previous report [39], germline ablation of FGFR4 caused no detectable changes on the tissue architecture or cellular relationships in the mammary fat tissue component, muscular component and lactiferous ducts, lobules or terminal units. Lactation, birth rate and survival of offspring were also similar to WT female mice (not shown). However, upon MMTV-promoter-driven TGFα overexpression, both KO-Tg and Tg female mice started to develop palpable breast tumors that disrupted the normal breast tissue structure 4 to 6 months after birth (Figure 1). There was no apparent phenotypic effect on male mice in either genetic group. Breast tumors were 70% cystic- and 30% solid-type. Most breast tumors were localized in the upper trunk of the body and more than 85% of tumors occurred in a single breast site. Quantitative analyses of palpable breast tumors along with microscopic pathological observations in both age-matched 218 Tg and 206 KO-Tg mice revealed that the Tg mice consistently had higher incidence of tumors and more rapid tumor progression than the KO-Tg mice (Figure 1). At 8 to 9 months, tumor incidence in Tg mice was 23 to 28% and the incidence in KO-Tg mice was about 5 to 8%. Tumor incidence reached a plateau of about 55 to 60% in the Tg group, which was only 17 to 22% in the KO-Tg group at 10 to 12 months (Figure 1). Toward the end of the observation period mandated by the animal protocol and limits on tumor burden, only mice from the KO-Tg group remained as a result of reduced tumor incidence and delayed tumor progression.

Foot and toe cysts were observed in both genotypes. Some mice with breast tumors had cysts, but not all mice with foot and toe cysts had palpable breast tumors. There was also spontaneous sebaceous gland hyperplasia in both groups evidenced as multiple raised white plaques or nodules on the abdomen. In contrast to breast tumors, the incidence of foot and toe cysts as well as the sebaceous gland hyperplasia was elevated in the KO-Tg mice relative to Tg mice. Greater than 90% incidence of foot and toe cysts and 24% incidence of sebaceous gland hyperplasia were observed in the KO-Tg mice compared to only 12 and 11%, respectively, in the Tg mice (not shown).

### The FGFR4 deficiency increases survival rate

**Figure 2:**
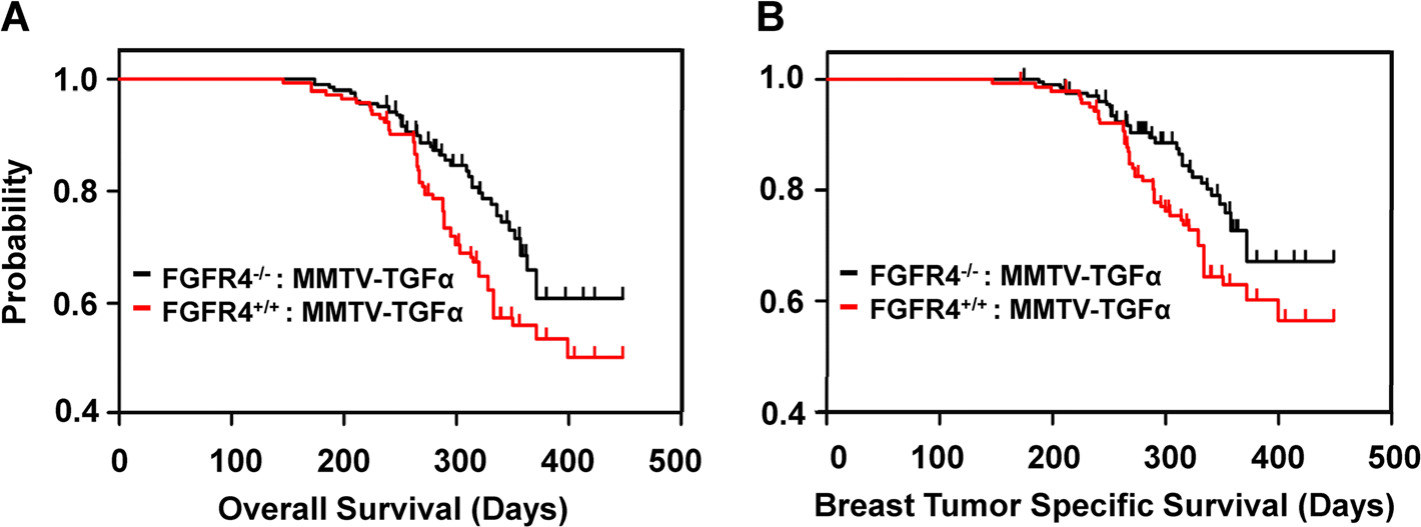
**Fibroblast growth factor receptor (FGFR)4 deficiency increased the survival of mice with breast tumor.** Life spans were scored in days as described in Methods. We monitored 245 Tg and 216 KO-Tg mice. The rates of overall **(A)** and post breast tumor diagnosis survival **(B)** in both the FGFR4-deficient and WT mice with TGFα overexpression were determined by Kaplan-Meier analysis. Tg, MMTV-TGFα transgenic; KO, FGFR4 knockout; WT, wild-type; TGFα, transforming growth factor alpha.

Breast cancer host survival rates were determined by the end-stage dictated by the extent of cancer burden that required sacrifice, or by their natural death as a result of mortal breast tumor burden and/or illnesses, such as foot and toe cysts and sebaceous gland hyperplasia. The overall survival rate of both genetic groups was defined as total months to sacrifice or natural death as a result of TGFα overexpression and/or FGFR4 deficit. The rate of breast tumor-specific survival was defined as the number of months until sacrifice or death after specific diagnosis of breast cancer. We analyzed 245 WT mice and 216 FGFR4-deficient mice, both expressing MMTV promoter-driven TGFα, using the Kaplan-Meier method. The overall survival (Figure 2A) and breast tumor-specific survival (Figure 2B) rates were both higher in the FGFR4-deficient TGFα transgenic mice than the FGFR4-WT TGFα transgenic mice.

### The FGFR4 deficiency delays pathological progression of breast cancer

**Figure 3:**
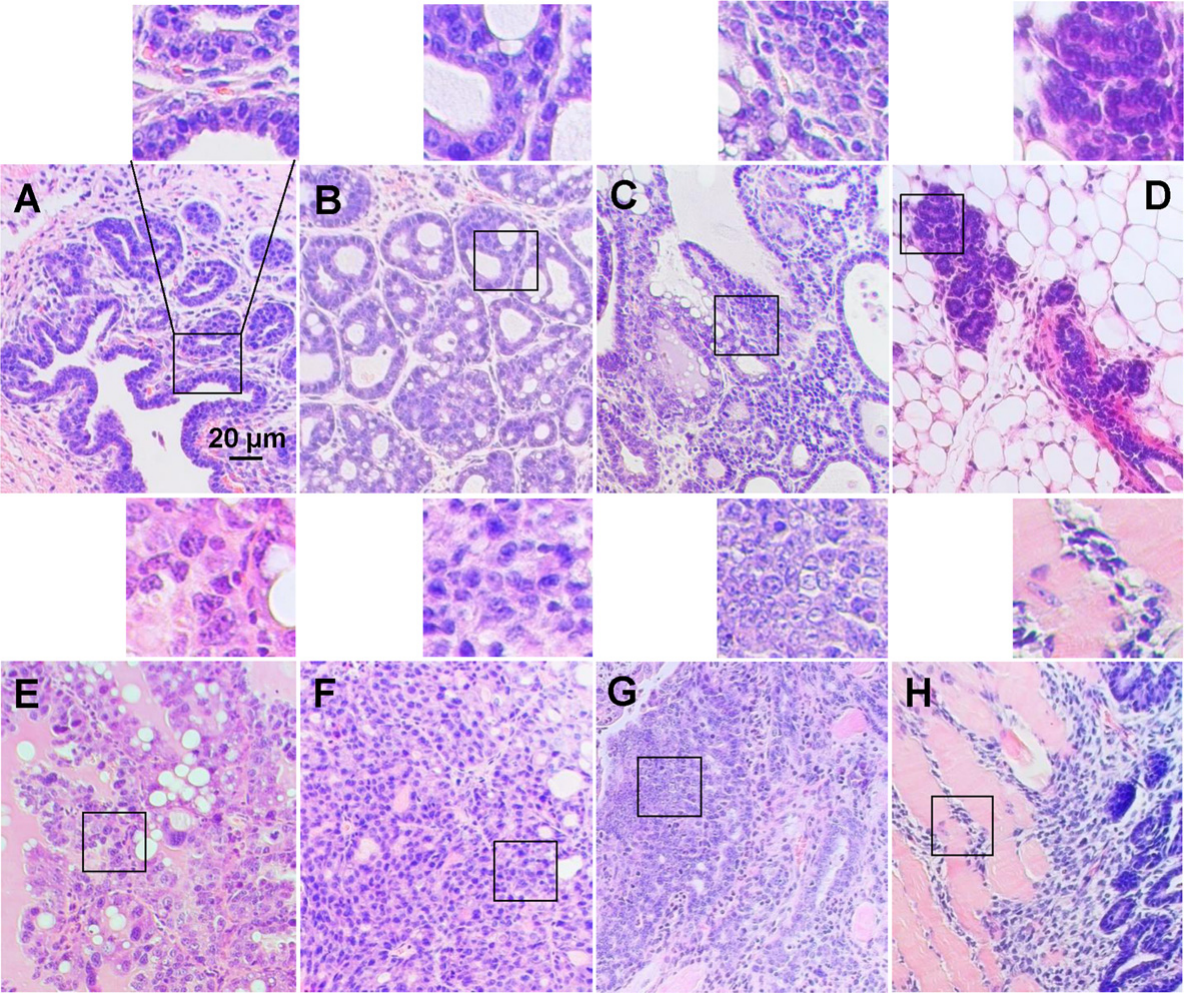
**Fibroblast growth factor receptor (FGFR)4 deficiency delayed pathological development of breast tumors.** Mammary gland lesions were identified in the breast tumor tissue sections from the MMTV-TGFα transgenic (Tg) and FGFR4^−/−^: MMTV-TGFα bigenic (KO-Tg) mouse groups and compared at a similar age of 6 months. Most of the breasts or palpable tumor nodes from the KO-Tg group were found in lower grades including the usual duct hyperplasia (UDH) **(A)**, lobular carcinoma *in situ* (LCIS) **(B)**, and duct carcinoma *in situ* (DCIS) **(C)**; while at a comparable age they were in higher grade of UDH **(E)**, invasive lobular carcinoma **(F)** and invasive duct carcinoma **(G**, **H)** in the Tg group. This is in contrast to breasts from wild-type mice with the typical duct and lobular structures surrounded by adipocytes **(D)**. Micrographs are representative of about 312 and 288 samples or sections from the Tg and KO-Tg groups, respectively.

**Figure 4:**
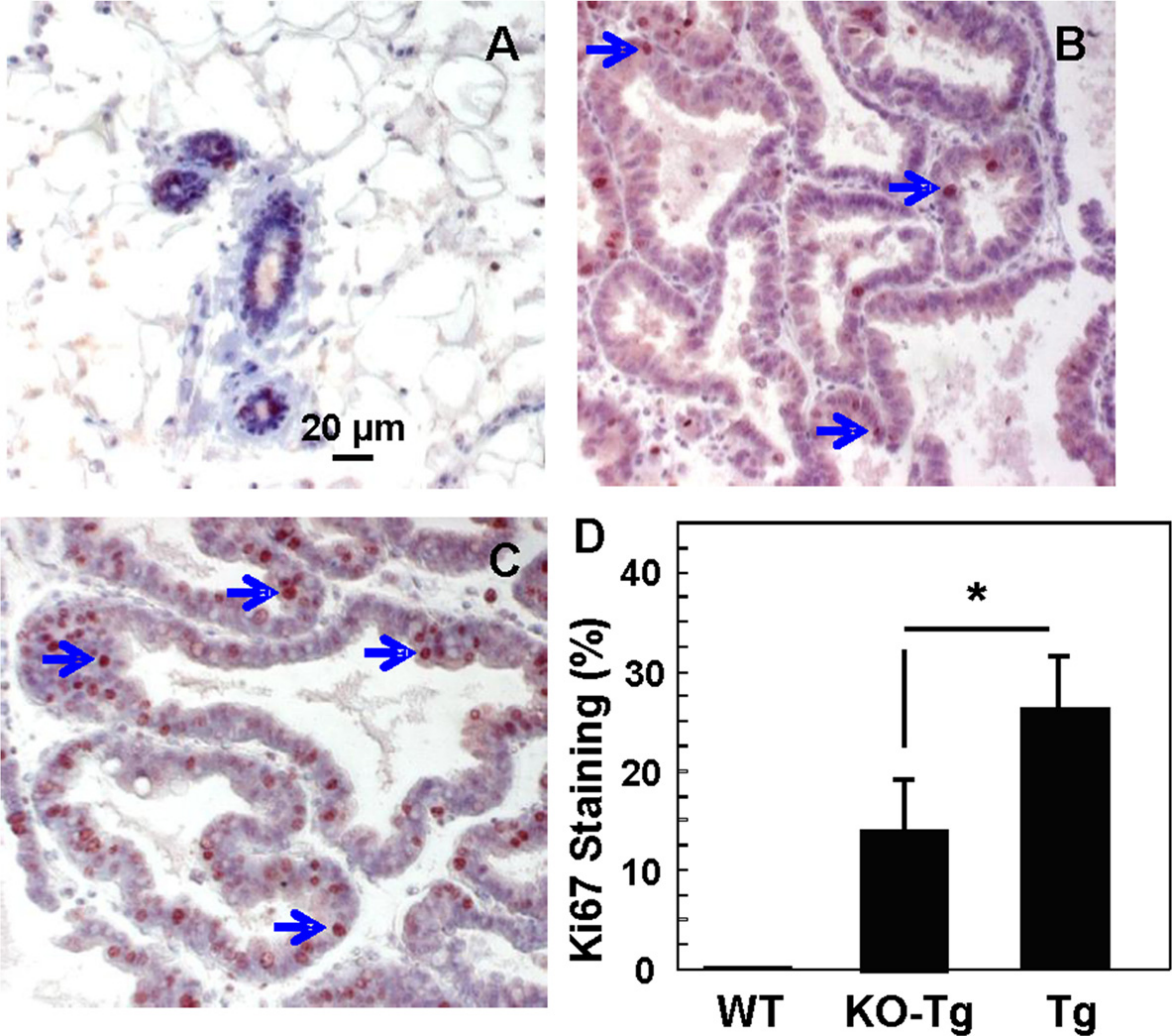
**Fibroblast growth factor receptor (FGFR)4 deficiency reduces mitoses in breast tumor foci.** The expression of Ki67 were assessed by immunohistochemical analyses of tissue sections from wild-type (WT) **(A)**, FGFR4^−/−^: MMTV-TGFα bigenic (KO-Tg) **(B)** and MMTV-TGFα transgenic (Tg) mice **(C)** (n = 12 for each group) at 6 months as indicated, and quantified by counting the positive dots in the populations of tumor cells in different random fields under the light microscope **(D)**. Data are expressed as percentage and means ± SD; ^*^P <0.05.

Age-matched tumor samples were collected from both groups. Serial sections of early breast tumor tissues at 4 to 8 months were graded for pathological stages in breast tumor development from mammary gland hyperplasia through ductal carcinomas *in situ* (DCIS) and lobular carcinomas *in situ* (LCIS) to invasive carcinoma. The pathologist was blinded to the genotypes of the tissue samples. Most of the breast or palpable tumor nodes from the KO-Tg group at 6 months were found predominantly in lower grades of the usual duct hyperplasia (UDH) (57%) (Figure 3A) and high-grade UDH (hUDH) (20%), whereas LCIS (Figure 3B) and DCIS (Figure 3C) were only 10 and 13%, respectively (Table 1). In contrast, tumors from the Tg group at the same age exhibited a higher grade of anatomopathological features. With a significant decrease in the grade of UDH to 16%, the hUDH (Figure 3E), LCIS and DCIS were significantly increased to 26, 23 and 28%, respectively, with the appearance of invasive lobular carcinoma (Figure 3F) and invasive ductal carcinoma (Figure 3G) (Table 1). These cellular/tissue abnormalities were in contrast to breasts from WT mice that exhibited typical duct and lobular structures with limited cellularity surrounded by adipocytes and stromal components (Figures 3D, 4A). Most tumors in both groups resembled the highly expanded tubular, adenoid cystic or papillary types shown in Figures 3A-C, 3E and 4B-C. Adenomyoepithelioma, myofibroblastic sarcoma, basal-like carcinoma, neuroendocrine carcinoma, glycogen-rich clear-cell carcinoma, phyllodes tumors, breast lymphatic invasion and liposarcoma appeared sporadically (data not shown). After 8 months, most of the palpable breast cancer nodes in Tg mice were either cystic or solid with over 65% containing poorly differentiated and highly invasive duct and lobular carcinomas or solid sheets of tumors of no specific type (Figure 3F, 3G, 3H). Among them, 34% of breast tumors exhibited highly disorganized infiltrating tumor cells indicated by easily identifiable muscle invasion (Figure 3H) (Table 1). In contrast, about 25% of the KO-Tg mice developed poorly differentiated carcinomas, and among them only 12% developed invasive carcinoma. No detectable metastasis in distal tissues or organs was evident in either genotype. The end-stage breast cancer samples harvested after mandatory sacrifice irrespective of the age exhibited no significant differences in tumor pathology despite the reduced tumor incidence and higher survival rate in the KO-Tg group. These results indicate that the absence of FGFR4 exerts its effect during early breast tumor progression.

**Table 1:** Effects of FGFR4 deficiency on the pathological development of mammary tumors*

**Age (months)**	**Genotype**	**UDH**	**hUDH**	**DCIS**	**LCIS**	**Undiff STS**	**IDC**	**ILC**
6	**KO**- **Tg**	**57**	**20**	**10**	**13**	**0**	**0**	**0**
6	**Tg**	**14**	**26**	**23**	**28**	**2**	**2**	**5**
8	**KO**-**Tg**	**11**	**27**	**16**	**21**	**13**	**5**	**7**
8	**Tg**	**6**	**10**	**8**	**11**	**31**	**15**	**19**

*Tissue sections from 24 mice each for the FGFR4^−/−^: MMTV-TGFα bigenic (KO-Tg) and MMTV-TGFα transgenic (Tg) mouse groups at 6 and 8 months were examined for the anatomopathological grades. The predominant pathological category for each section was recorded and the percentage score of each pathological category in total sections analyzed was reported. Three sections for each sample were analyzed. UDH, usual duct hyperplasia; DCIS, ductal carcinomas *in situ*; LCIS, lobular carcinomas *in situ*; hUDH, high-grade UDH; IDC, invasive ductal carcinoma; ILC, invasive lobular carcinoma; Undiff STS, undifferentiated solid tumor sheet.

Consistent with the delay in breast cancer development indicated by the pathological analyses, the mitotic index indicated by IHC staining for Ki67 was reduced by 15% in 6-month breast tumor foci from the KO-Tg compared to age-matched Tg mice (Figure 4B, 4C, 4D). As expected, breast ducts and lobular units in samples from normal WT mice exhibited few mitotic figures (Figure 4A, 4D). There was also a similar 22% reduction in the BrdU staining in the KO-Tg breast tumors compared to the Tg tumors (data not shown). This further suggests an effect of FGFR4 deficiency on early development of breast tumorigenesis.

### Negligible expression of FGFR4 in breast and lack of effect on Her2 activation

**Figure 5:**
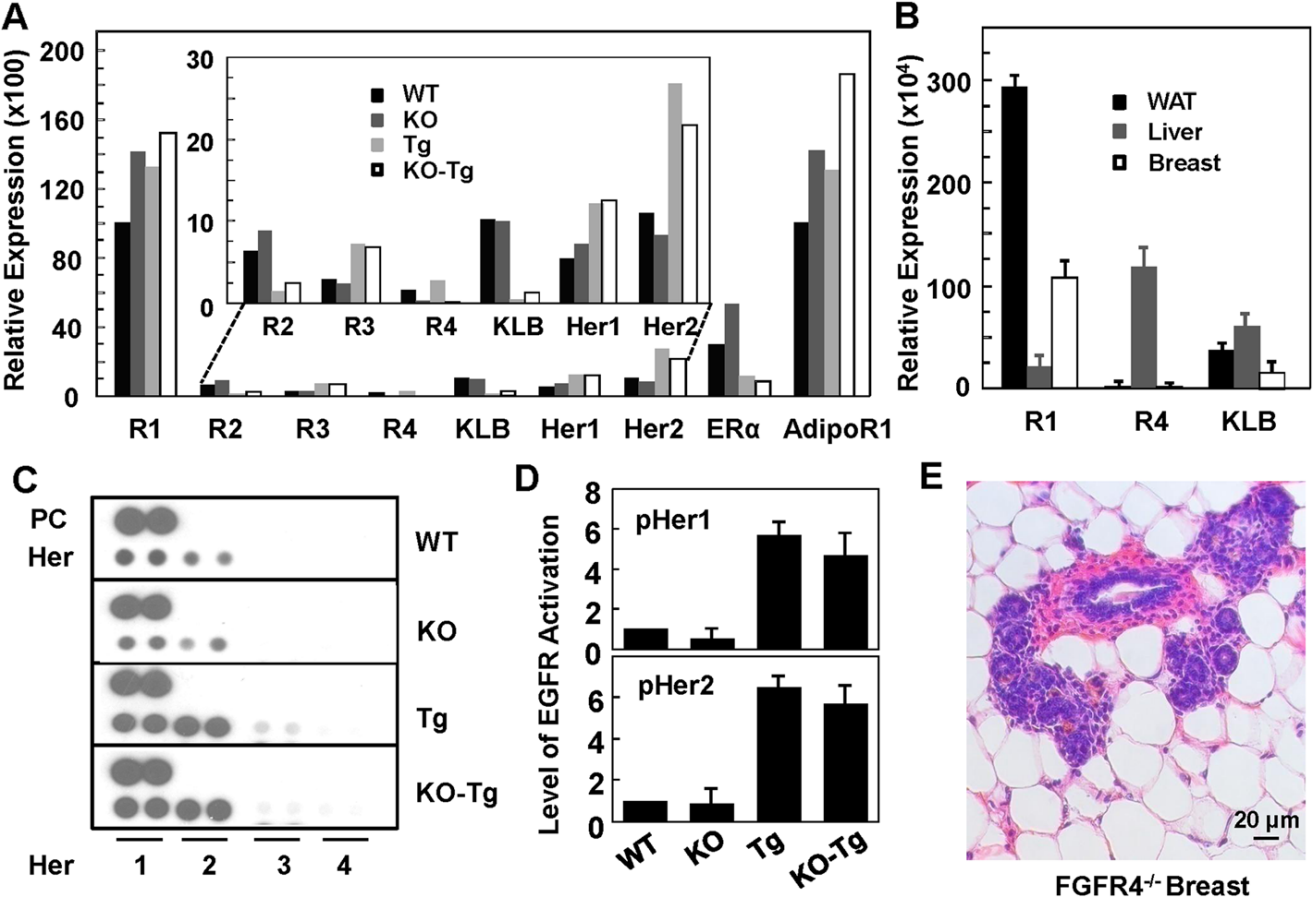
**Insignificant effects of fibroblast growth factor receptor (FGFR)4 deficiency on human epidermal growth factor receptor (Her) and FGFR expression, Her2 activation and cellularity of the mouse breasts. (A)** The expression of FGFR1/2/3, Her1/2, betaKlotho (KLB), estrogen receptor (ER)α and adiponectin receptor (AdipoR)1 in the breasts and tumors from the wild-type (WT), FGFR4 knockout (KO), MMTV-TGFα transgenic (Tg) and FGFR4^−/−^: MMTV-TGFα bigenic (KO-Tg) mice at 6 months was analyzed by quantitative PCR (see Methods). mRNA levels were normalized to β-actin and expressed as fold change relative to that of FGFR1 in WT breasts, which was assigned an arbitrary unit of 100. **(B)** Comparative expression of FGFR1, FGFR4 and KLB in the white adipose tissue (WAT), liver and breast were analyzed by quantitative PCR, and expressed as changes relative to β-actin levels in the respective tissues. Indicated data are means ± SD, *P* <0.05 (n = 6/group). **(C)** Antibody array for a panel of activated kinases was done (see Methods) using tissue extracts from six mouse breasts in each of the WT, KO, Tg and KO-Tg groups. Representative micrographs of the dot blot strips for the four members of the Her family are shown. PC, positive antibody control. **(D)** Quantitative densitometric analyses of phosphorylated Her1 (pHer1) and Her2 from **(C)** shown as means ± SD, *P* <0.05 (n = 3). **(E)** Representative section of the mammary tissues from the KO mice at 6 months showing no change in the cellularity and structure of the mammary tissue.

We investigated mechanisms by which the FGFR4 deficit led to the delay in breast cancer progression driven by the overexpression of TGFα. Quantitative PCR analyses at the mRNA levels for members of the EGFR family, Her1 to 4, and FGFR family, FGFR1 to 4, revealed that the FGFR1, Her1 and Her2 are the major isotypes of the FGFR and EGFR family in the breast, respectively (Figure 5A). They were increased to a low but statistically significant extent in the breast tumor tissues in both KO-Tg and Tg groups (Figure 5A). After normalization to β-actin, the expression level of FGFR4 was the lowest or negligible, which was about 140-fold lower than FGFR1 and about 17-fold less than Her2 (Figure 5A). Both expression levels for FGFR2 and FGFR3 were also low but considerably exceeded that of FGFR4 in breasts of FGFR4-WT mice. The expression of FGFR4 was 152-fold lower in breast tissue than the liver, which is the only known tissue expressing functional FGFR4 at a high level (Figure 5B). In addition, the expression of FGFR4 in the white adipose tissue (WAT) is also negligible as compared to FGFR1 and KLB. These results indicate that FGFR4 expression and therefore, its tyrosine kinase activity, is likely insignificant within the cells of the breasts and breast tumors.

TGFα, whose overexpression drives the breast tumors in the mouse model studied here, is closely related to EGF and an activating ligand for the proto-oncogene Her2, also called Neu or ErbB-2, a member of the EGFR family [51]. Phospho-tyrosine (active) kinase antibody array analyses on breast and breast tumor tissues revealed that the activities of both EGFR (Her1) and Her2 were enhanced about 7.5-fold in the TGFα transgenic mice relative to the non-transgenic controls (Figure 5C, 5D). However, there was no apparent difference between the FGFR4^−/−^ and FGFR4^+/+^ groups together with TGFα transgene or without it. The expression and activity of Her3 and Her4 were also negligible (Figure 5A). Thus the FGFR4 deficiency has no direct and significant effect on tumor-associated levels of the EGFR family members Her1 and 2, two of which likely underlie the TGFα-driven tumors.

KLB, the co-receptor required for the metabolic activities of FGFR4, is expressed in the breast at a level of about 3- to 5-fold less than visceral WAT and the liver (Figure 5B). KLB is localized in the stromal fraction, most of which is fat-like tissue, but not the luminal epithelial compartment (Additional file 1: Figure S1A, orange staining). It is significantly reduced in breasts with developing tumor nodes due to loss of adipose tissue, and is not present in the tumor foci (Figure 5A, Additional file 1: Figure S1B). In contrast, FGFR1, the partner of KLB activated by FGF21, is widely expressed in adipocytes, normal luminal epithelium and tumor cells (Additional file 1: Figure S1C, S1D, orange staining). The expression of estrogen receptor alpha (ERα) was also reduced during breast tumorigenesis driven by TGFα (Figure 5A).

### Alterations of systemic metabolic factors

Consistent with a negligible level of expression and lack of significant effect on Her1/2 activity and with previous reports, there was no significant change in the breast tissue architecture and function due to the FGFR4 deficiency in the WT mice (Figures 5E, 3D and 4A) [30,39]. We thus turned to the systemic metabolic activities of FGFR4 as a potential source of the negative impact on breast tumor progression. Elevation of bile acids through abrogation of FGFR4-KLB in the liver is the early and most well-documented effect of a genome-wide deficiency in FGFR4 [30]. Analysis of fecal content of bile acids indeed revealed an elevation in the KO-Tg mice, similar to the FGFR4^−/−^ -alone mice (Additional file 1: Figure S2A). Previously reported changes in serum glucose, lipids and insulin sensitivity caused by FGFR4 deficiency were also recapitulated in the KO-Tg mice (Additional file 1: Figure S2B, S2C, S2D). FGFR4 deficiency had little effect on body weight (Additional file 1: Figure S2E).

**Figure 6:**
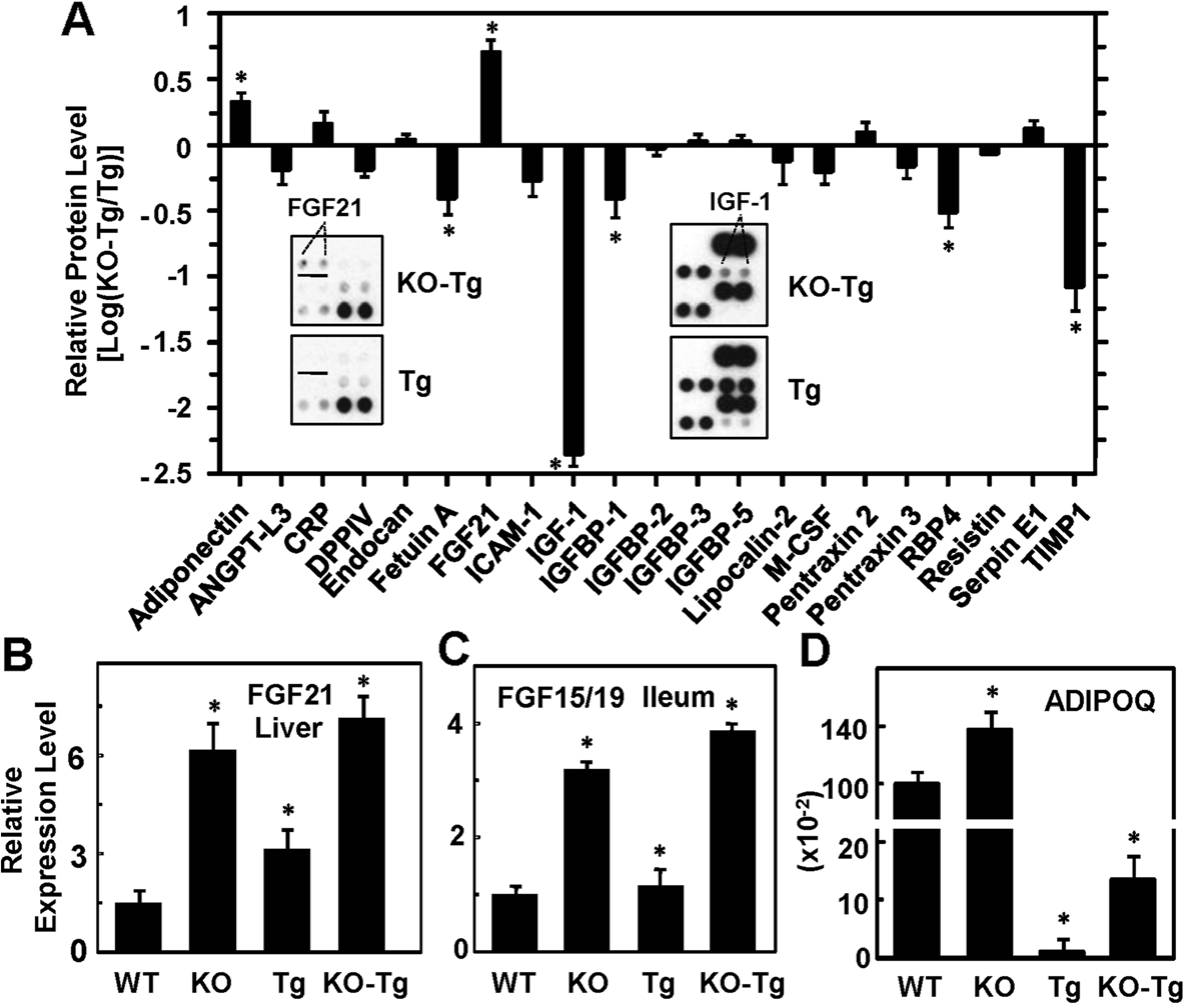
**Effects of fibroblast growth factor receptor (FGFR)4 deficiency on systemic adipokine levels in tumor**-**bearing mice. (A)** Antibody-based array analyses. The panel of adipokines as indicated was measured simultaneously in sera from the MMTV-TGFα transgenic (Tg) and FGFR4^−/−^: MMTV-TGFα bigenic (KO-Tg) mice (n = 8 for each group) at 6 months. Changes of adipokine levels were expressed as log of the densitometric ratio of KO-Tg to Tg. Data are means ± SD; *P* <0.05. **(B**-**D)** Changes in expression of liver FGF21, ileal FGF15 and breast adiponectin (ADIPOQ) in the wild-type (WT), FGFR4 knockout (KO), Tg and KO-Tg mice at 6 months were determined by quantitative PCR. Results were normalized to the β-actin levels in the respective samples and then expressed as fold change relative to that of WT, which is assigned as an arbitrary unit of 1 or 100. Data are means ± SD, ^*^P <0.05 (n = 6 for each group).

Examination of circulating adipokines by antibody array in age-matched KO-Tg and Tg mice revealed a 5-fold increase in the systemic level of endocrine FGF21 in the FGFR4^−/−^ mice relative to that in the FGFR4^+/+^ mice (Figure 6A and inset). Among a panel of other adipokines analyzed, adiponectin levels were increased more than 2-fold, and Fetuin A, insulin-like growth factor (IGF)-1, IGF binding protein (IGFBP)-1, retinol binding protein (RBP)4 and tissue inhibitor of metalloproteinase (TIMP)1 were decreased 2.5-, 25.0-, 2.5-, 3.0-, and 12-fold, respectively, in the FGFR4^−/−^ mice bearing breast tumors (Figure 6A and *inse*t). These significant changes were further confirmed by quantitative PCR. FGF21 expression in the liver, the predominant production site of endocrine FGF21, was consistently elevated by 2.5- to 4.0-fold in the FGFR4^−/−^ background (Figure 6B). An increase of about 3- to 4-fold in the expression of endocrine FGF15, the mouse counterpart of human FGF19, in the ileum was also observed in the FGFR4^−/−^ mice (Figure 6C). Hepatic FGF21 is the major eFGF that activates FGFR1-KLB in adipocytes, and ileal FGF15/19 is the primary source of the factor that activates FGFR4-KLB in hepatocytes and secondarily FGFR1-KLB in adipocytes.

### Changes in expression of genes involved in breast tissue metabolism

Circulating FGF21 and enterohepatic FGF19 are potent systemic metabolic regulators, and at elevated levels in response to stress, they normalize local and systemic metabolic parameters by targeting the adipose tissue and liver through FGFR1 and FGFR4, respectively, in partnership with KLB [10,17,18,52]. We showed by analysis of early gene responses to FGF21 that FGF21 also impacts the breast fat tissue directly [17], owing to the expression of KLB in adipose compartment together with FGFR1 (Figure 5A, 5B, Additional file 1: Figure S1A, S1C). Recent analyses using our adipocyte-specific FGFR1-deficient mice along with KLB-deficient mice [13,18,20] has made it clear that the adipocyte FGFR1-KLB complex specifically mediates the great majority of systemic metabolic as well as anti-obesogenic and anti-diabetic effects of FGF21. This indicates that the adipocyte is the specific target of endocrine FGF21, which underlies its therapeutic activities against obesity and diabetes that are also risk factors for some cancers. Although hepatocytes are the primary target of FGF15/19, the endocrine factor may also act on the FGFR1-KLB partnership in adipocytes similar to FGF21. FGFR4 is not expressed in the breast that is comprised mostly of adipocytes (fat) and its deficiency elevates both FGF21 and FGF15/19 and other adipokines (Figures 5A, 5B and 6). Thus the mechanisms underlying the delay of TGFα-driven breast cancer development by the FGFR4 deficit may be related to metabolic activities of FGF21/19 that cause systemic metabolic programming that in turn impacts local metabolic, and therefore, cellular effects such as the decrease in mitoses (Figure 4). We thus proceeded to perform analyses of the effect of the FGFR4 deficiency on expression of major regulatory and enzyme genes involved in the pathways of adipogenesis, fatty acid metabolism, glucose metabolism and mitochondrial function in normal breast and breast tumor.

An increased expression of adiponectin (ADIPOQ) in breast tissues in all FGFR4-ablated mice mirrored that in the circulation (Figure 6D, 6A). Even in tumor foci where ADIPOQ was drastically depressed more than 100-fold, ADIPOQ was increased 11-fold by FGFR4 deficiency (Figure 6D). Expression of adiponectin receptor 1 (AdipoR1) was as high as FGFR1 and increased in tumors hosted in the FGFR4-deficient mice (Figure 5A).

Other diverse genes exhibiting either up- or down-regulation consistently across age-matched (8 months) pairs of WT and KO, and Tg and KO-Tg tissues, were summarized according to those potentially contributing to the delay of breast tumorigenesis by FGFR4 deficiency. We predict that these changes may be the major contribution and/or consequence of the FGFR4 deficiency and the related elevation in the systemic FGF21 and FGF19 in mammary tumorigenesis.

Adipose tissue is not only the fat energy repository, but also a dynamic endocrine organ important for hormone and adipokine secretion that mediates cross-talk between adipocytes and multiple organs [50,53,54] (Figure 6A). Adipose tissue dysfunction contributes to obesity and metabolic syndrome, which are risk factors for breast cancer [21,22,24]. Analyses of expression of 84 key genes involved in adipogenesis and adipocyte functions in the breast and mammary tumor tissues from WT and KO, and from Tg and Tg-KO indicated that the FGFR4 deficiency impacts the adipogenesis and adipocyte functions related to adipokine secretion and lipid metabolism in both breast and breast tumor tissues. FGFR4 ablation in general across each group decreased the expression of Srebp1, Cebp delta, Dlk1, Klf2, Irs2, E2F, CDK4, CCND1, Jun, Src and Wnt5b, while increased the expression of adiponectin, adipsin, HSL, PPAR alpha, Cdkn1a, Fabp4 (aP2) and UCP1 (Table 2). These changes have been implicated in growth, differentiation and maintenance of adipocytes and thus may perform the same functions in the breast adipose tissue compartment and modulation of lipolysis and lipogenesis in the breast. These may also indirectly affect development of breast tumor cells in the breast microenvironment. Notably, some of the changes were related to inhibition of cell proliferation. The increases in adiponectin and adipsin, and changes of systemic levels of other adipokines further indicate the changes of secretory function of the adipose component in the breast, which may impact breast epithelial tumors expressing adipokine receptors such as AdipoR1 at an early stage of development.

**Table 2:** **Effects of FGFR4 deficiency on adipogenesis**, **adipose secretory function and metabolic pathways in breasts and breast tumor**

**Proteins**	**WT**	**KO**	**Tg**	**KO**-**Tg**
**1. Adipokines and adipose enzymes**:				
Adiponectin	1	1.64 (0.23)*	0.01 (0.002)	0.11 (0.02)
Adipsin	1	3.32 (0.17)	0.12 (0.03)	0.43 (0.06)
Leptin	1	0.55 (0.04)	0.003 (0.0001)	0.05 (0.004)
Lipoprotein lipase	1	0.92 (0.17)	0.08 (0.02)	0.25 (0.06)
Hormone sensitive lipase	1	2.02 (0.31)	0.11 (0.02)	0.31 (0.03)
**2. PPAR gamma targets**:				
Fabp4	1	1.62 (0.26)	0.04 (0.01)	0.15 (0.03)
FASN	1	1.01 (0.23)	0.14 (0.03)	0.35 (0.04)
IRS2	1	0.12 (0.03)	1.84 (0.35)	0.27 (0.08)
Glut4	1	0.66 (0.12)	0.05 (0.003)	0.18 (0.04)
SREBP1	1	0.27 (0.06)	1.97 (0.29)	0.33 (0.04)
**3. Adipogenesis regulation**:				
(1) Pro-adipogenesis				
Cyclin D1	1	0.46 (0.10)	6.61 (0.29)	3.42 (0.18)
Cdk4	1	0.43 (0.08)	5.14 (0.37)	2.23 (0.19)
Cebpd	1	0.24 (0.05)	7.26 (0.48)	2.46 (0.15)
E2F1	1	0.23 (0.03)	2.16 (0.36)	0.71 (0.11)
Jun	1	0.56 (0.08)	2.93 (0.26)	1.52 (0.30)
Sfrp5	1	0.55 (0.10)	0.01 (0.002)	0.04 (0.005)
Src	1	0.19 (0.03)	4.72 (0.51)	2.31 (0.38)
Wnt5b	1	0.35 (0.06)	3.01 (0.23)	1.37 (0.29)
(2) Anti-adipogenesis				
Cdkn1a (p21)	1	1.65 (0.18)	2.44 (0.14)	3.71 (0.26)
Dlk1	1	0.31 (0.04)	1.03 (0.13)	0.12 (0.03)
Klf2	1	0.007 (0.001)	1.23 (0.16)	0.004 (0.0005)
(3) Others				
Ppara	1	7.33 (0.47)	0.31 (0.05)	1.36 (0.17)
Ucp1	1	177.1 (1.57)	0.12 (0.02)	9.94 (1.03)

^*^m*RNA* levels were normalized to β-actin levels in the respective samples and then expressed as fold changes relative to that of the wild-type (WT) breasts, which is assigned as an arbitrary unit of 1. Data are mean (SD): *P* <0.05 (n = 4 for each group). FGFR, fibroblast growth factor receptor; KO, FGFR4 knockout mice; Tg, MMTV-TGFα transgenic mice; KO-Tg, FGFR4^−/−^: MMTV-TGFα bigenic mice.

The FGFR4 deficiency causes mildly elevated levels of systemic lipids accompanied by a mild obese phenotype [36]. Alterations in the expression of genes involved in fatty acid and lipid metabolism are associated with metabolic syndrome or metabolic disorders. They are also risk factors for multiple diseases, including obesity and diabetes as well as cancer [55,56]. Aberrant oxidation of fatty acids generates reactive oxygen species (ROS) that cause cell damage and tissue inflammatory response. Analyses of 84 key genes and other components involved in the regulation and enzymatic pathways of fatty acid and triacylglyceride metabolism revealed the upregulation of Acly, Acot3, Acsl5, Acsm3, Cpt1b, Cpt2, Fabp4, Gyk, Gpd2, HSD17B4, Lipe and Slc27a4, and downregulation of Acox2, Fabp5 and Slc27a5 (Table 3) due to FGFR4 deficiency across the breast and tumor tissues in each group. Acot3, Acox2 and HSD17B4 are peroxisomal enzymes, Acsl5 and Acsm3 are mitochondrial enzymes, and Slc27a4 and Slc27a5 are ER-localized enzymes involved in the metabolism (oxidation and synthesis) of medium-chain, and long-chain to very-long-chain fatty acids, including cholesterol/bile acids. Slc27a5 is involved in both bile acids and very-long-chain fatty acid synthesis. Cpt1b and Cpt2 are members of the carnitine O-palmitoyltransferase family involved in the net transport of long-chain fatty acyl-CoAs from the cytoplasm into the mitochondria for beta-oxidation. Fabp4 binds both long-chain fatty acids and retinoic acid and delivers them to their cognate receptors in the nucleus, such as PPARs. Mitochondrial Gyk and Gpd2 are key enzymes in the regulation of glycerol uptake and metabolism. These results indicate that the FGFR4 deficiency in large part alters fatty acid and lipid catabolism and energy metabolism primarily in mitochondria, peroxisomes and the ER in breast tissues. Some of these changes have been reported as contributing factors in the pathology of not only obesity and associated diabetes but also cancer.

**Table 3:** Effects of FGFR4 deficiency on fatty acid metabolic pathways in breasts and breast tumor

**Proteins**	**WT**	**KO**	**Tg**	**KO**-**Tg**
**1. Fatty acid metabolism**				
Acot3	1	3.63 (0.23)*	1.41 (0.11)	7.92 (0.44)
Acsl4	1	1.25 (0.17)	26.51 (1.47)	52.33 (3.74)
Acsl5	1	6.52 (0.51)	1.36 (0.14)	5.27 (0.48)
Acsm3	1	16.45 (0.77)	0.87 (0.09)	5.16 (0.42)
Aldh2	1	2.14 (0.13)	2.72 (0.26)	8.03 (0.58)
Acly	1	3.43 (0.37)	1.64 (0.25)	4.32 (0.31)
Acox2	1	0.46 (0.08)	24.76 (0.87)	11.16 (0.58)
HSD17B4	1	1.88 (0.33)	1.62 (0.24)	3.45 (0.41)
**2. Fatty acid transport**				
Cpt1b	1	6.92 (0.71)	0.13 (0.02)	0. 72 (0.14)
Cpt2	1	2.82 (0.17)	0.63 (0.09)	3.64 (0.24)
Fabp4	1	1.53 (0.31)	0.05 (0.01)	0.26 (0.02)
Fabp5	1	0.02 (0.003)	0.61 (0.05)	0.04 (0.01)
Slc27a4	1	1.91 (0.35)	0.28 (0.04)	0.64 (0.08)
Slc27a5	1	0.16 (0.04)	2.31 (0.19)	0.74 (0.12)
**3. Triacylglycerol Metabolism**				
GyK	1	3.97 (0.28)	10.34 (0.72)	33.59 (0.65)
Gpd2	1	9.92 (0.56)	0.96 (0.22)	4.03 (0.35)
Lipe	1	2.30 (0.15)	0.16 (0.03)	0.43 (0.12)
Lpl	1	0.9 (0.17)	0.12 (0.02)	0.30 (0.03)

^*^Data are mean (SD): p <0.05 (n = 4 for each group). FGFR, fibroblast growth factor receptor; KO, FGFR4 knockout mice; Tg, MMTV-TGFα transgenic mice; KO-Tg, FGFR4^−/−^: MMTV-TGFα bigenic mice.

The genome-wide FGFR4 deficiency causes moderate, but significant changes in glucose and insulin sensitivity. Changes in glucose metabolism are common in cancer progression that most often exhibits increased aerobic glycolysis and decreased oxidative phosphorylation even in the presence of sufficient oxygen to actively supply both energy and substrates of biosynthesis [57,58]. We further analyzed 84 key genes involved in the regulation and enzymatic pathways of glucose metabolism in the breast and breast tumor tissues. Our analyses revealed the upregulation of fructose-1,6-bisphosphatase (Fbp)2, phosphoenolpyruvate carboxykinase (Pck)1, pyruvate dehydrogenase kinase (Pdk)4 and glycogen synthetase (Gys)2, and downregulation of 2-phospho-D-glycerate hydrolyase (Eno)3, phosphoglycerate mutase (Pgam)2, mitochondrial glucokinase (Gck or HK4), the catalytic subunit of glucose-6-phosphatase (G6pc) and glycogen phosphorylase (Pygm) (Table 4). Fbp2 is a regulatory enzyme in gluconeogenesis that catalyzes the hydrolysis of fructose 1,6-bisphosphate to fructose 6-phosphate. Pck1, also called PEPCK1, is the main control point in gluconeogenesis by catalyzing the formation of phosphoenolpyruvate from oxaloacetate. PDK4 inhibits the mitochondrial pyruvate dehydrogenase complex, therefore decreases the oxidation of pyruvate in mitochondria to acetyl-CoA. Gys2 is the rate-limiting step in the synthesis of glycogen. Eno3 plays a role in converting phosphoglyceric acid to phosphoenolpyruvic acid in the glycolytic pathway. Pgam2 catalyzes the reversible reaction of 3-phosphoglycerate to 2-phosphoglycerate in the glycolytic pathway. Gck phosphorylates glucose to produce glucose-6-phosphate as the first step in the glucose metabolic pathway. G6pc hydrolyzes glucose-6-phosphate to glucose in the ER. Pygm is an enzyme involved in glycogenolysis. The changes in these genes observed in the FGFR4 deficient breast tissues cluster around those associated with negative effects on glycolysis and glucose oxidation as well as promotion of glucose storage. These collective changes are also generally consistent with the delay of tumorigenesis.

**Table 4:** Effects of FGFR4 deficiency on glucose metabolic pathways in breasts and breast tumor

**Proteins**	**WT**	**KO**	**Tg**	**KO-Tg**
**1. Glucose metabolism:**				
Eno3	1	0.18 (0.04) ^*^	0.10 (0.02)	0.03 (0.002)
Pgam2	1	0.22 (0.03)	0.11 (0.02)	0.05 (0.01)
Gck	1	0.45 (0.07)	0.41 (0.07)	0.07 (0.02)
Fbp2	1	2.23 (0.37)	0.07 (0.01)	0.55 (0.04)
G6pc	1	0.04 (0.003)	1.43 (0.11)	0.13 (0.03)
Pck1	1	3.39 (0.44)	0.01 (0.001)	0.12 (0.02)
Pdk4	1	6.86 (0.57)	0.15 (0.03)	1.02 (0.14)
**2. Glycogen metabolism**				
Gys2	1	2.79 (0.19)	0.02 (0.003)	0.12 (0.03)
Pygm	1	0.22 (0.03)	0.08 (0.01)	0.04 (0.002)

^*^Data are mean (SD): p <0.05 (n = 4 for each group). FGFR, fibroblast growth factor receptor; KO, FGFR4 knockout mice; Tg, MMTV-TGFα transgenic mice; KO-Tg, FGFR4^−/−^: MMTV-TGFα bigenic mice.

As some of the genes in the forgoing analyses were related to mitochondrial energy metabolism, we further analyzed 84 key and related genes involved in the biogenesis and function of mitochondria. Tumor cells often exhibit mitochondrial dysfunction that is associated with increased glycolysis, oxidative stress and ROS damage. Deregulation of these processes is a major pathological factor in metabolic syndrome, obesity and cancer progression [59-61]. In addition to some of those described above, we consistently found the upregulation of Cpt1b and mitochondrial uncoupling protein (UCP)1 as well as mitofusin (Mfn)2, and downregulation of NAMPT, Slc25a5 and Slc25a21 (Table 5). UCP1 (Slc25a7) separates oxidative phosphorylation from ATP synthesis with energy dissipated as heat. Mfn2 encodes a mitochondrial membrane protein that participates in the maintenance and operation of the mitochondrial network. It may be also a hyperplasia suppressor and a risk gene for obesity. NAMPT is the rate-limiting enzyme in the mammalian nicotinamide adenine dinucleotide biosynthesis (NAD) salvage pathway. It is overexpressed in multiple cancers, and inhibition of NAMPT significantly suppresses tumor cell growth. Slc25a5, adenine nucleotide translocator 2 (Ant2), catalyzes the exchange of cytoplasmic ADP with mitochondrial ATP across the mitochondrial inner membrane. Suppressed expression of this gene has been shown to induce apoptosis and inhibit tumor growth. Slc25a21, 2-oxodicarboxylate carrier, transports C5-C7 oxodicarboxylates across the inner membranes of mitochondria including 2-oxoadipate, 2-oxoglutarate, adipate and glutarate, which are common intermediates in the catabolism of lysine, tryptophan and hydroxylysine in mammals. Slc25a24 is another Ca^2+^-dependent mitochondrial carrier protein for ATP-Mg/Pi. Overall, the changes in expression of these genes appear to be largely related to mitochondrial biogenesis, energy production and homeostasis. Consistent with the tumor-delaying effect of the FGFR4 deficiency, some of these changes largely have been associated with tumor development.

**Table 5:** Effects of FGFR4 deficiency on mitochondrial biogenesis and energy metabolism in breasts and breast tumor

**Proteins**	**WT**	**KO**	**Tg**	**KO**-**Tg**
Cpt1b	1	6.92 (0.24)^*^	0.13 (0.02)	0.72 (0.04)
Mfn2	1	1.92 (0.34)	0.26 (0.03	0.49 (0.03)
NAMPT	1	0.22 (0.01)	7.64 (0.65)	2.33 (0.37)
Slc25a5	1	0.44 (0.16)	3.92 (0.43)	1.67 (0.23)
Slc25a21	1	0.31 (0.05)	0.60 (0.04)	0.09 (0.02)
Slc25a24	1	0.57 (0.07)	4.14 (0.29)	1.91 (0.13)
Ucp1	1	170.5 (1.57)	0.17 (0.02)	8.54 (0.45)

^*^Data are mean (SD): p <0.05 (n = 4 for each group). FGFR, fibroblast growth factor receptor; KO, FGFR4 knockout mice; Tg, MMTV-TGFα transgenic mice; KO-Tg, FGFR4^−/−^: MMTV-TGFα bigenic mice.

### Changes in the expression of inflammatory factors

**Figure 7:**
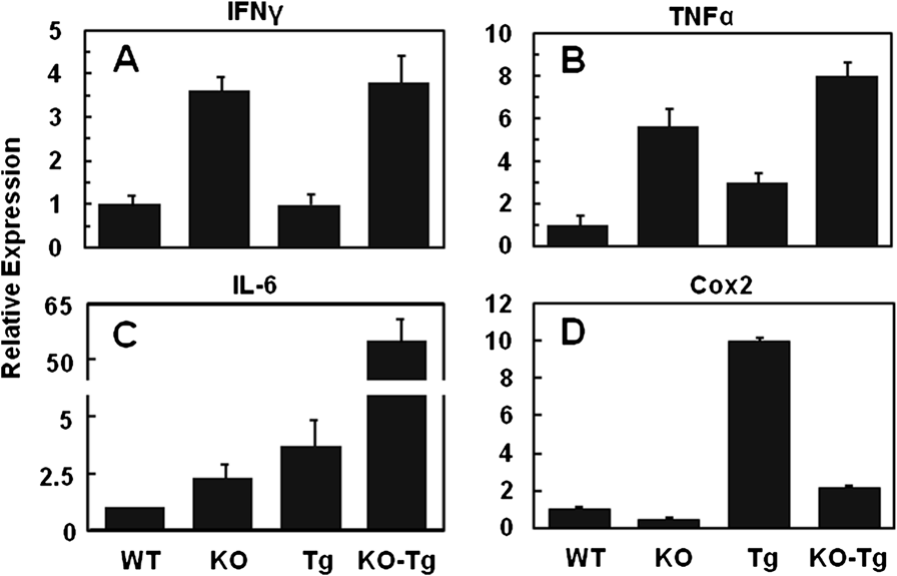
**Upregulation of some inflammatory genes due to fibroblast growth factor receptor (FGFR)4 deficiency.** Changes in expression of IFNγ **(A)**, TNFα **(B)**, IL-6 **(C)** and Cyclooxygenase (Cox)2 **(D)** in the breast and breast tumor tissues from wild-type (WT), FGFR4 knockout (KO), MMTV-TGFα transgenic (Tg) and FGFR4^−/−^: MMTV-TGFα bigenic (KO-Tg) mice at 6 months were determined by quantitative PCR. mRNA levels were normalized to β-actin levels in the respective samples and then expressed as fold changes relative to that of the WT breast, which is assigned as an arbitrary unit of 1. Data are means ± SD; *P* <0.05 (n = 4 for each group).

Several metabolic genes described above that exhibited changes due to the FGFR4 deficiency are also associated with the response of peroxisomes-ER-mitochondria system upon metabolic alterations and stress. This is consistent with a large number of observed cystic breast tumors as well as an increased rate of foot cyst and sebaceous gland hyperplasia triggered by the FGFR4 deficiency. This indicates a possible inflammatory response contributing to these types of abnormalities. We thus selectively analyzed the changes of IFNγ, TNFα and IL-6 across each of the WT and KO, and the Tg and KO-Tg groups (Figure 7). FGFR4 ablation led to increases of IFNγ, TNFα and IL-6 more than 3.5-, 5.0- and 4.0-fold, respectively (Figure 7A, 7B and 7C). The overexpression of TGFα also led to increases of TNFα and IL-6 (Figure 7B and 7C). The combination of FGFR4 deficiency and TGFα overexpression further increased expression levels that were particularly notable in the case of IL-6 (Figure 7C). In contrast, the FGFR4 deficiency decreased the expression of Cyclooxygenase (Cox)2 (Figure 7D).

### Inhibition of mammary tumor cells by targeting NAMPT

**Figure 8:**
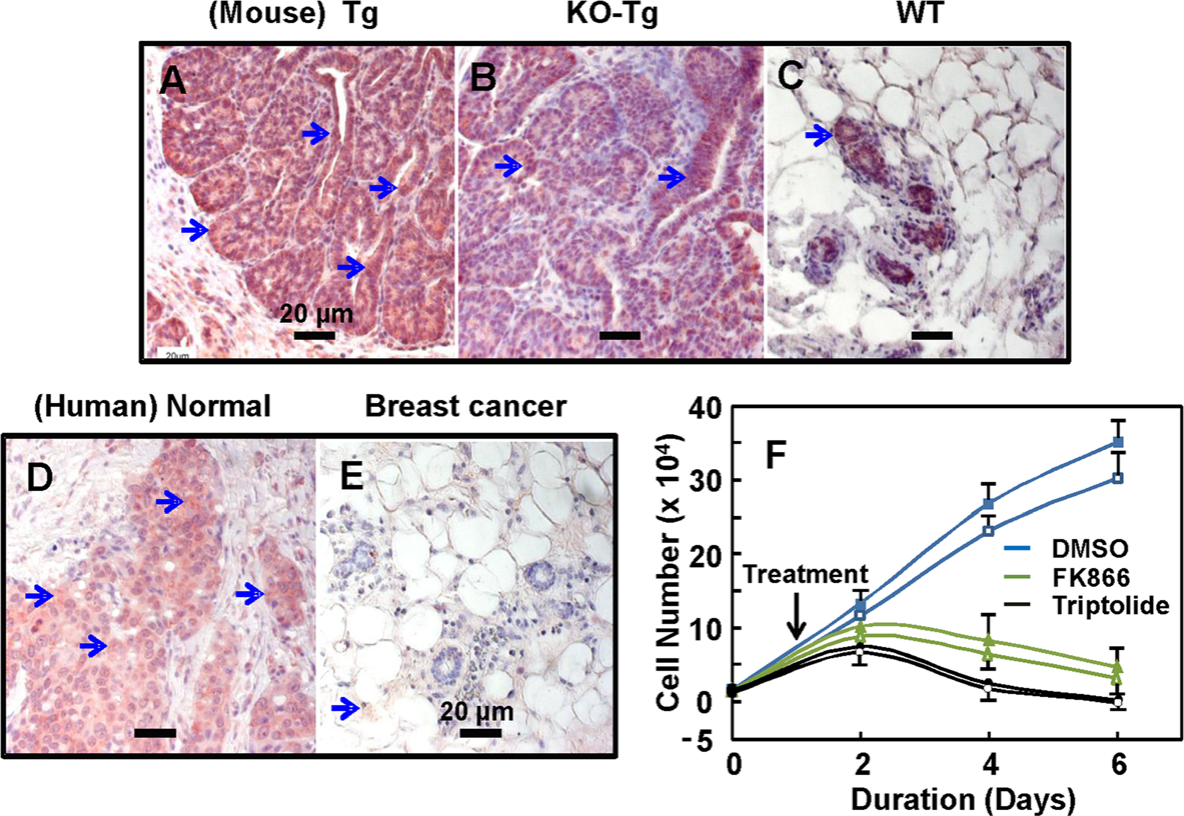
**Inhibition of mammary tumor cell growth by targeting nicotinamide phosphoribosyltransferase (NAMPT).** Tissue sections from mouse breast tumor nodes in MMTV-TGFα transgenic (Tg) mice **(A)**, FGFR4^−/−^: MMTV-TGFα bigenic (KO-Tg) mice **(B)** and normal breast **(C)**, or from human breast tumor **(D)** and matched normal breast **(E)** were analyzed for NAMPT expression (blue arrow, orange staining) by IHC, examined under microscope and representative sections were shown. Tumor cells were isolated from established solid tumor foci in Tg and KO-Tg mice, cultured for one day and then treated with vesicle dimethyl sulfoxide (DMSO), 5 μM FK866 or 5 μM Triptolide for 5 days. Treatment with FK866, a catalytic inhibitor for NAMPT, significantly reduced the population growth of primary tumor cells **(F)**. Light blue line: vesicle DMSO; green line: 5 μM FK866; black line: 5 μM Triptolide as positive control; Filled shapes (square, triangle and circle): tumor cells from Tg mice; open shapes: tumor cells from KO-Tg mice.

We observed in metabolic pathways by quantitative PCR analyses that NAMPT is overexpressed in breast tumor tissue and FGFR4 deficiency downregulates the expression. To determine whether this occurs in tumor foci, we performed IHC analyses on tissue sections from mice and human patients. Results indicate that mouse NAMPT is highly expressed in the luminal epithelium-derived tumor cells with highly expanded tubular, adenoid cystic and papillary tumor tissue structures (Figure 8A, orange staining), and that FGFR4 deficiency attenuates the NAMPT levels (Figure 8B, 8C). The cytosolic staining was also uniformly high in the tumor foci from 87% of 48 breast cancer patients analyzed (Figure 8D), but was weak in the matched phenotypically normal breast tissues and the adipose and stromal tissues surrounding tumor nodules (Figure 8D, 8E).

Our results indicate that NAMPT may be a downstream target for further reduction of breast tumorigenesis. We used a catalytic inhibitor of NAMPT, FK866, which was shown to deplete energy reserve in metabolically active tumor cells and induce tumor cell death, to treat the isolated tumor cells from solid tumor nodes. FK866 at 5 μM led to significant inhibition of cell population growth (Figure 8F). On day 5 of treatment, FK866 reduced the cell populations to 13 and 10% in the Tg and KO-Tg groups, respectively, of the untreated cells. As a comparison, a highly cytotoxic compound extracted from Tripterygium, Triptolide, also exhibited potent suppressive effect (Figure 8F, Additional file 1: Figure S3A-S3C). Tumor sphere formation in a serum-free condition is indicative of ability of single primary tumor cells to clonally expand and repopulate tumors. Isolated primary tumor cells exhibited 0.47 and 0.25% efficiency from 3,000 cells/well in a cell-culture well with a culture surface of 10 cm^2^ from the Tg and KO-Tg tumors, respectively, in forming visible tumor spheres (Additional file 1: Figure S3D, S3E); the presence of 5 μM FK866 blocked formation of the tumor sphere (not shown).

## Discussion

Alterations in local and systemic glucose, lipid and energy metabolism and mitochondrial function are often associated with progression of multiple types of cancer, including breast cancer [21,24,55-57,59,62]. In this report, we evaluated the local and systemic metabolic effects resulting from a germline deficiency of metabolic regulator FGFR4 [30] on breast cancer development in the MMTV-TGFα mouse model [8,9]. The central function of the WT FGFR4 tyrosine kinase is metabolic regulation in the liver where it is highly expressed [30,37]. It is, however, expressed at negligible levels in breast [38]. In contrast to other receptor tyrosine kinases with growth-promoting effects involved in tissue and cellular structure development, adult damage repair and tumorigenesis, such as FGFR1 [1,2,6,63], overexpression, or deletion of FGFR4 caused no defects in embryonic development and adult tissue and cellular homeostasis beyond metabolic homeostasis [30,36,39]. In particular, there were no detectable effects on mammary cellular and tissue structure, lactation, birth rate and survival of offspring beyond tumorigenesis caused by overexpression of transgenic TGFα. Even though FGFR4 is not at play in breast cells in general, we have shown that it affected systemic metabolic parameters and factors that impact the breast through the non-canonical metabolic functions of eFGF. Notably, deficiency of FGFR4 significantly upregulated hepatic and systemic FGF21, which is a stress-induced hormone mediating communication with adipocytes via FGFR1-KLB to trigger adipocyte signals and metabolites that in turn impact liver and other tissues [13,50]. Breast is comprised of mostly adipose tissue, which is responsive to changes in systemic metabolism. The adipose compartment creates a microenvironment symbiotic with breast epithelial cells in ducts and lobules with large impact on functions and pathology including development of breast tumors. Similar to adipocytes in peripheral fat depots, KLB and FGFR1 are co-expressed in the fat in breast (Figure 5, Additional file 1: Figure S1), which enables the response of breast to FGF21 and potentially other factors that target adipocytes [17]. The metabolic alterations and upregulation of FGF21 by loss of FGFR4, the fatty nature of breast and the lack of mammary expression of FGFR4 thus underlie the model used in this study for evaluating the systemic and microenvironmental metabolic effects on breast epithelial carcinogenesis.

Indeed, by combining TGFα overexpression in breast epithelial cells and the FGFR4 deficiency outside the breast, we found that ablation of FGFR4 reduced TGFα-driven breast cancer incidence, delayed breast cancer progression and improved host survival. This coincided with reduced local tumor cell proliferation. Although the deletion of FGFR4 had no significant effect on Her2 activity [64] stimulated by overexpression of TGFα, the deficiency significantly elevated hepatic and systemic FGF21, ileal FGF15/19 and serum adiponectin. In contrast, several other adipokines including Fetuin A, IGF-1, IGFBP-1, RBP4 and TIMP1 were depressed in the FGFR4-deficient animals. The changes of these serum adipokines and factors triggered by the FGFR4 deficiency are generally consistent with the observed net tumor suppressive effect, and thus support our notion that the effect of FGFR4 deficiency on delaying breast cancer progression lies in the alteration of metabolic pathways governed by eFGF signaling and other systemic factors.

Adiponectin is an early response effecter in the FGF21-KLB-FGFR1 pathway in adipocytes [18,65,66]. It is an adipose hormone involved in the suppression of metabolic diseases, such as obesity and non-alcoholic fatty liver disease (NAFLD), by reducing lipogenesis and systemic lipid levels and enhancing fatty acid oxidation through acting on AdipoR1/2 [22,53,67,68]. It is suggested to also have anti-proliferative, anti-inflammatory, and therefore, antitumor effects associated with metabolic disorders. We show here that the adiponectin receptor adipoR1 is highly expressed in both breast and breast epithelial tumors. Thus both adiponectin of systemic origin from distal fat depots and from local breast adipocytes under the control of FGF21 could play a major role directly on breast tumor epithelial cells and their microenvironment. IGF-1 is a hepatic hormone and promotes cell growth and carcinogenesis [69,70]. TIMP1, an inhibitor of the matrix metalloproteinases (MMPs), has also been shown to promote tumor progression in numerous tissues [71]. Collectively, these specific positive and negative alterations of local microenvironmental and systemic adipokines and factors are consistent with the delay of breast tumorigenesis.

**Figure 9:**
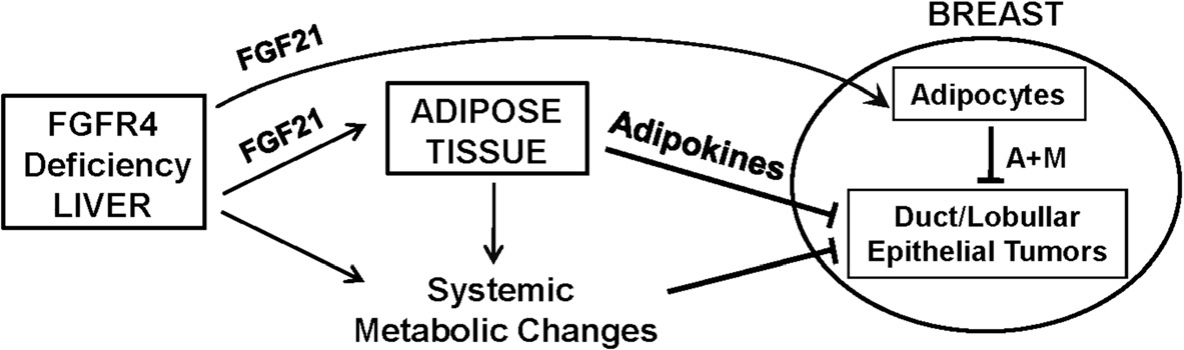
**Possible mechanisms of inhibition of mammary tumorigenesis by fibroblast growth factor receptor (FGFR)4 deficiency.** Deficiency of hepatic metabolic regulator FGFR4 has little effect on breast cellularity and function where FGFR4 is not at play. The deficiency causes systemic metabolic changes and constitutive elevation of expression of systemic FGF21 which is normally elevated only during diverse hepatic stress conditions that are disease-suppressive. FGF21 specifically acts on adipocytes both in fat depots and breast. Both circulating and breast microenvironmental secretory products from adipocytes affect cellular and metabolic behavior of breast epithelial cells. Systemic changes in metabolism governed by hepatocyte FGFR4-betaKlotho (KLB) and both systemic and microenvironmental changes governed by adipocyte FGFR1-KLB impact breast parenchymal cell behavior and tumor progression. A + M: adipokines and metabolic effects.

FGF21 and FGF19 are potent regulators of glucose, lipid and energy metabolic homeostasis and suppress obesity and diabetes through targeting adipose tissue and liver [14,18,20,26]. These metabolic effects occur without a same extent of growth and proliferation-promoting activity characteristic of canonical FGFs such as FGF1 and FGF2 [11,14]. We confirmed our prediction that the breast adipose compartment is also a target of systemic FGF21 [17]. The upregulation of Cpt1, Pck1, PPARα and Ucp1 in addition to adiponectin in breast tissue is consistent with previous studies in the independent adipose tissue and adipocytes, which showed that FGF21 activates the adipose FGFR1-KLB complex leading to significant changes in the expression of these metabolic genes and regulators [18,72]. Therefore, these results support the notion that FGF21 and possibly FGF19/15 as well, are top candidates underlying changes in metabolic pathways and concurrent delay of breast cancer development observed here (Figure 9). They not only modulate metabolic pathways in peripheral fat depots that affect the breast systemically, but also directly act on breast adipocytes with major microenvironmental impact on mammary epithelial cells during development of mammary tumor. These activities of FGF21 and possibly FGF19 on adipocytes that elicit tumor suppressive metabolic signals appear sufficient to override tumor-promoting effects of elevated bile acids, inflammation and mild metabolic abnormalities elicited by the FGFR4 deficiency.

Consistently, we further showed the downregulation of several cell proliferation-promoting genes such as CCND1, CDK4, E2F, Jun, NAMPT, Src and Wnt5b and inflammatory Cox2, and upregulation of proliferation-inhibitory genes such as Cdkn1a and Mfn2. These changes are likely not limited to only adipose tissue and adipogenesis. They are in line with the upregulation of adiponectin and downregulation of systemic factors such as IGF-1 and TIMP1 that otherwise promote cell proliferation. NAMPT as a downstream target of FGFR4 deficiency and FGF21 elevation is highly expressed in breast tumor foci but attenuated by the FGFR4 deficiency. Inhibition of NAMPT by FK866 suppressed the growth of isolated tumor cells from both Tg and KO-Tg mice and the formation of tumor spheres from single tumor cells, an indicator of the presence of cells with the ability to expand and repopulate tumors (Figure 8, Additional file 1: Figure S3). This is consistent with other studies in many other types of cancer [73,74], in which NAMPT is overexpressed or mutated. Downregulation or blockade of the NAD^+^ generation led to cessation of cell proliferation or apoptotic cell death. Enhanced NAMPT activity insures a constant energetic growth capacity and malignant behavior by constantly replenishing the NAD^+^ pool that critically links glycolysis, the tricarboxylic acid (TCA) cycle, oxidative phosphorylation, ATP flux, inflammation and redox regulation.

Furthermore, we found the upregulation of Acot3, Acsl5, Acsm3, HSD17B4, HSL and CPT1/2 that indicate reduced lipogenesis or enhanced lipolysis and fatty acid oxidation. Upregulation of Acly, Fbp2, Gys2, Pck1 and Pdk4 and downregulation of Eno3, Pgam2, Gck and Pygm indicate an inhibition of glycolysis, the hallmark of tumorigenesis [57,58,75,76]. Acsl5, Acsm3, Cpt1/2, Gpd2, Mfn2, Slc25a5, Slc25a21, Slc25a24 and Ucp1 are localized in mitochondria. Changes of these mitochondrial genes are consistent with changes in fatty acid and lipid catabolism and with changes of enzymes localized in the peroxisomes and ER. Defects in biogenesis, glucose/fatty acid oxidation and energy production of mitochondria lead to changes in glycolysis, oxidative stress and ROS damage that are factors common to obesity and tumorigenesis [21,23,24,55,59,61,77,78]. Changes of these metabolic enzymes and regulators are consistent with systemic elevation and anti-obesogenic functions of FGF21 and FGF19 that translate into a delay of breast tumorigenesis.

We showed previously that FGF21 is induced in the liver by diverse transcriptional regulators in response to a wide range of stress conditions that include obesity, fatty liver disease and carcinogenesis [13,50]. Hepatic FGF21 controls the hepatocyte to adipocyte communication arm of a hepatic-adipose tissue partnership to meet stress challenges through restoring metabolic homeostasis [13,50]. Based on our current results, breast is also within this communication axis. Moreover, since breast is essentially an adipose organ, its non-adipocyte components are also the recipients of this axis within the local breast tissue microenvironment. The effects of the FGFR4 deficiency on breast tumors are most likely through these systemic indirect metabolic effects that modify the local adipose tissue microenvironment and epithelial compartment in breast duct and lobular structures and the developing malignant cells. These indirect effects are likely mediated in large part by elevated FGF21 and FGF19, which regulate pathways related to adipogenesis and adipose function, lipid and glucose metabolism, peroxisomal and ER metabolic stress, and mitochondrial function and energy metabolism as reported here and by others [14,18]. We therefore propose that in respect to breast, this hepatic-breast adipose communication axis may have evolved as an overall tumor suppressive influence in the local breast microenvironment. This is likely interwoven with the benefit of the general axis to the organism through normalizing and reprogramming metabolic parameters and pathways during stress conditions, in particular tumorigenic metabolic stress (Figure 9).

This study extends our discovery that hepatic FGFR4 signaling underlies control of cholesterol to bile acid metabolism by showing the local and systemic effects on tumor development in a tissue where FGFR4 is normally not directly at play. The corollary of the tumor-suppressive effects of the FGFR4 deficiency in the breast is that chronic activity of hepatic FGFR4 and its systemic endocrine effects may contribute to promotion of breast tumors via the inverse of the changes in systemic adipokines and factors and local metabolic reprogramming we reported here. Our study implicates for the first time a potential microenvironmental metabolic role of the adipocyte compartment in breast tumor progression in response to eFGF/FGFR signaling. The notable rise in FGF21 that is specific for adipocyte FGFR1-KLB and correlates with the delay in breast tumor progression suggests that FGF21 and its agonists may be of similar benefit for control of breast tumor progression, as they are for alleviation of obesity and diabetes via the adipocyte compartment. Furthermore, our study provided potential new targets, such as FGF21, adiponectin, NAMPT and Acly, for suppressing breast tumor malignancy concurrently with inhibition of FGFR.

## Conclusions

Here we showed that systemic and local metabolic alterations caused by a deficiency of the metabolic regulator FGFR4 reduce TGFα-induced tumor incidence and progression in breasts where FGFR4 expression is negligible. The FGFR4 deficiency causes increases in systemic factors, including the adipocyte-targeting hepatic stress hormone FGF21 and ileal FGF15 (human FGF19), accompanied by increases in the tumor-suppressive adipokine adiponectin and decreases in tumor-promoting adipokines IGF-1 and TIMP1 that are controlled by anti-obesogenic FGF21. Changes in major metabolic pathways related to adipocyte function, lipid and glucose metabolism and mitochondrial function within the breast and tumor tissues are coincident with the delay of mammary tumorigenesis. They suggest novel roles of FGF21 signaling and metabolic reprogramming in suppressing mammary tumors that rise from luminal epithelial cells in the ducts and lobules surrounded mostly by FGF21-responsive adipocytes. Our results provide new insights into the impact of microenvironmental and systemic metabolic reprogramming by endocrine FGF signaling on tumorigenesis in the breast where adipocytes are a major symbiotic component.

ADIPOQ: adiponectin; AdipoR1: adiponectin receptor 1; BrdU: 5-Bromo-2′-deoxyuridine; BSA: bovine serum albumin; Cox2: cyclooxygenase-2; DCIS: ductal carcinomas *in situ*; DMEM: Dulbecco’s modified Eagle’s medium; eFGF: endocrine fibroblast growth factor; EGFR: epidermal growth factor receptor; Eno3: 2-phospho-D-glycerate hydrolyase; Fbp2: fructose-1,6-bisphosphatase 2; FGFR: fibroblast growth factor receptor; FGF21: fibroblast growth factor-21; G6pc: catalytic subunit of glucose-6-phosphatase; Gck: mitochondrial glucokinase HK4; Gys2: glycogen synthetase; Her2; human epidermal growth factor receptor-2; H&E: hematoxylin and eosin; HS: heparan sulphate; hUDH: high-grade usual duct hyperplasia; IFN: interferon: IGF1: insulin-like growth factor-1; IL6: interleukin 6; IHC: immunohistochemistry; IPITT: intraperitoneal insulin tolerance test; KL: Klotho; KLB: betaKlotho; KO: FGFR4 knockout C57BL/6 J mice; KO-Tg: FGFR4^−/−^: MMTV-TGFα bigenic mice; LCIS: lobular carcinomas *in situ*; Mfn2: mitofusin 2; NAMPT: nicotinamide phosphoribosyltransferase; OGTT: oral glucose tolerance test; PBS: phosphate-buffered saline; Pck1: phosphoenolpyruvate carboxykinase 1 (PEPCK); PCR: polymerase chain reaction; Pdk4: pyruvate dehydrogenase kinase 4; Pgam2: phosphoglycerate mutase-2; Pygm: glycogen phosphorylase; RBP4: retinol binding protein-4; ROS: reactive oxygen species; TIMP1: tissue inhibitor of metalloproteinase-1; Tg: MMTV-TGFα transgenic mouse; TGFα: transforming growth factor α; TNF: tumor necrosis factor; UCP1: mitochondrial uncoupling protein 1; UDH: usual duct hyperplasia; WT: wild-type.

## Competing interests

All authors declare that they have no competing interests.

## Authors’ contributions

YL, WM, SY and ML participated in the design of study. CY, MY, CJ and YL performed research. YL, CY, WM, JLA and SY analyzed data. YL and WM wrote the manuscript. All authors read and approved the final manuscript.

## Supplementary Material

Additional file 1 ***Deficiency of metabolic regulator FGFR4 delays breast cancer progression through systemic and microenvironmental metabolic alterations *** **by Luo Y, et al.** Click here for file
