# Supplementary material for: Deficiency of metabolic regulator FGFR4 delays breast cancer progression through systemic and microenvironmental metabolic alterations
Source: Cancer Metab. 2013 Nov 25;1:21. doi: 10.1186/2049-3002-1-21 (PMC4178208; doi:10.1186/2049-3002-1-21)
Supplement: Additional file 1 — Deficiency of metabolic regulator FGFR4 delays breast cancer progression through systemic and microenvironmental metabolic alterations by Luo Y, et al. [file 2049-3002-1-21-S1.docx]

**Supplementary Data**

“Deficiency of metabolic regulator FGFR4 delays breast cancer progression through systemic and microenvironmental metabolic alterations” by Luo Y., et al.

**Figure S1 Expression of KLB and FGFR1 in the breasts and breast tumor foci.** Tissue sections from phenotypically normal breasts of KO mice (**A, C**) and from tumor-bearing breasts of KO-Tg mice (**B, D**) at 6 month were analyzed for KLB (**A, B**) and FGFR1 (**C, D**) expression by immunohistochemistry [50]. Results indicate that KLB is expressed only in the stromal adipose tissue compartment (blue arrow), not in the luminal epithelium (**A**) and tumor cells (**B**) (yellow arrow). In contrast, FGFR1, which requires KLB for FGF21 activity, is expressed in adipose tissue compartment (purple arrow) in both KO breasts (**C**) and KO-Tg breast tumor (**D**), as well as the luminal epithelium (**C**) and tumor cells (**D**) (green arrow). Breasts and tumor tissues from WT and Tg mice have a similar expression pattern for KLB and FGFR1.

**Figure S2 FGFR4 deficiency-dependent metabolic changes are present in the tumor-bearing mice.** (**A**) Changes in fecal bile acid levels in the Tg and KO-Tg mice as compared to those of the WT and KO mice at 6 month. Fecal bile acid levels were measured as described in the Methods and expressed as means ± sd, * p<0.05 (n=6 for each group). (**B**) Mild glucose intolerance. Glucose (1g/kg body weight) was administered by gastric gavage to the Tg and KO-Tg mice after an overnight fasting. Blood samples were collected and measured before and 15, 30, 60 and 120 min after delivery of the glucose load. Data are means ± sd, p<0.05 (n=12 for each group). (**C**) Mild increase in blood triglycerides (TG). FGFR4 deficiency resulted in an increase of about 30% in serum TG level in both KO and KO-Tg mice. Overexpression of TGFα had little effect. Data are means ± sd, p<0.05 (n=12 for each group). (**D**) Mild insulin insensitivity. Insulin (0.75 units/kg body weight) was injected intraperitoneally into Tg and KO-Tg mice after fasting for 5 hours. Blood samples were collected and measured before and 15, 30, 45, 60, 90and 120 min after insulin administration. Data are means ± sd, p<0.05 (n=12 for each group). (**E**) FGFR4 deficiency has little effect on body weight. Body weights of the Tg (n=130) and KO-Tg (n=165) mice were monitored at the indicated time.

**Figure S3 Inhibition of mammary tumor cell growth by targeting NAMPT.** Tumor cells were isolated from solid tumor foci in KO-Tg mice, cultured for one day and then treated with vesicle DMSO (**A**), 5 µM FK866 (**B**) and 5 µM Triptolide (**C**) for five days. Cell populations were examined under microscope and representative views were shown (x 100). Treatment with FK866, a catalytic inhibitor for NAMPT, significantly reduced survival and population growth of primary tumor cells as compared to the untreated or treated with Triptolide. The ability to form tumor sphere from a single cell was examined for the isolated primary tumor cells from Tg (**D**) and KO-Tg (**E**) mice as described in Methods (x 20).
